# Microbial metabolite-driven immune reprogramming in tumor immunotherapy: mechanisms and therapeutic perspectives

**DOI:** 10.3389/fimmu.2025.1603658

**Published:** 2025-10-17

**Authors:** Yao Lu, Huiping Yuan, Shaojie Liang, Debing Li, Pengfei Jiang, Xian Wang, Ke Zhang, Dechun Liu

**Affiliations:** ^1^ Department of Thoracic Surgery, The First Affiliated Hospital, and College of Clinical Medicine of Henan University of Science and Technology, Luoyang, China; ^2^ Department of Endocrinology and Metabolism, The First Affiliated Hospital, and College of Clinical Medicine of Henan University of Science and Technology, Luoyang, China; ^3^ Department of Gastrointestinal Surgery, The First Affiliated Hospital, and College of Clinical Medicine of Henan University of Science and Technology, Luoyang, China

**Keywords:** microbial metabolites, tumor immunotherapy, immune regulation, tumor microenvironment, immune checkpoint inhibitors, immunotherapy resistance

## Abstract

The gut microbiome critically regulates antitumor immunity through its metabolic byproducts, which serve as pivotal mediators of host-microbe crosstalk in tumor immunotherapy. This review synthesizes cutting-edge evidence on how microbial metabolites—including short-chain fatty acids (SCFAs), tryptophan derivatives, and bile acids—reprogram immune cell dynamics and remodel the tumor microenvironment (TME). Mechanistically, metabolites such as butyrate and indole-3-propionic acid (IPA) enhance immune checkpoint inhibitor (ICI) efficacy by epigenetic modulation or metabolic reprogramming. Conversely, kynurenine (a tryptophan metabolite) and secondary bile acids drive resistance by polarizing macrophages toward an immunosuppressive phenotype or exhausting cytotoxic T cells. Metabolite-targeted interventions (such as probiotics, dietary modulation, and engineered microbes) show synergistic potential with ICIs, but require resolution of causal inference limitations, interindividual variability, tumor-context specificity, and dose optimization. Precision microbiome engineering, guided by multi-omics profiling and artificial intelligence, may unlock personalized strategies to overcome immunotherapy resistance.

## 1 Introduction

The microbiome comprises a diverse array of microbial communities residing internally and on the host’s surface, critically influencing host health and disease trajectories (1, 2). Through their metabolites, the microbiome modulates immune responses and tumor progression (3, 4). Tumor immunotherapy—particularly immune checkpoint inhibitors (ICIs)—has demonstrated paradigm-shifting clinical efficacy. However, this therapeutic strategy faces numerous challenges, including variability in patient responses and the immunosuppressive nature of the tumor microenvironment (TME) (5, 6). Microbial metabolites exhibit direct oncotoxic effects and synergistically amplify immunotherapy efficacy through immune reprogramming, establishing their potential as novel adjuvants. However, the therapeutic promise of microbial metabolites is tempered by fundamental challenges: (1) contradictory immunomodulatory effects observed across experimental contexts; (2) overreliance on correlative human data without mechanistic validation; (3) clinical failures of metabolite-targeted agents despite strong preclinical rationale; and (4) interindividual variability in microbial metabolic capacity. This review critically evaluates these complexities, establishing frameworks to reconcile conflicting data and prioritize translationally viable metabolites.

## 2 Microbial metabolites: mechanistic classifications and context-dependent signaling networks

### 2.1 Types and sources: core mechanistic frameworks

Microbial metabolites encompass a structurally diverse array of bioactive molecules synthesized through fermentation, respiration, and secondary metabolism, including short-chain fatty acids (SCFAs), tryptophan (Trp) derivatives, bile acids, polyamines, polysaccharides, and lipopolysaccharides (LPS) (4, 7). These metabolites function as pleiotropic regulators of immunity and metabolism through three conserved mechanistic networks: (1) receptor-mediated signaling (e.g., G protein-coupled receptors [GPCRs], nuclear receptors), (2) epigenetic modulation (e.g., histone deacetylase [HDAC] inhibition), and (3) metabolic reprogramming (e.g., energy substrate provision, redox balance) (8, 9). Their production is dynamically regulated by host diet, microbial community structure, and environmental cues, creating a complex interactome that dictates functional outcomes (9, 10).

Critically, the immunomodulatory effects of microbial metabolites are determined by context-dependent variables including: (1) concentration gradients (e.g., micromolar vs. millimolar ranges), (2) tissue-specific receptor expression (e.g., G protein-coupled receptor [GPR]43 on T cells vs. epithelial cells), (3) metabolic microenvironment (e.g., glucose availability, redox state), and (4) host genetic background. This context dependency explains the frequently observed functional duality (immunostimulatory vs. immunosuppressive) that characterizes many microbial metabolites and necessitates mechanistic rather than descriptive classification.

### 2.2 SCFAs: GPCR signaling and epigenetic modulation

SCFAs—predominantly acetate, propionate, and butyrate—are microbial fermentation products of indigestible dietary fibers, with butyrate (1–10 mM colonic concentrations) serving as the primary energy substrate for colonocytes (11). These metabolites exert their effects through dual mechanisms: (1) GPCR activation on immune and epithelial cells, and (2) HDAC inhibition leading to epigenetic reprogramming, with their net effect determined by concentration gradients and target cell type (12). SCFAs coordinate intestinal barrier integrity through enhanced tight junction expression while regulating inflammatory homeostasis via modulation of nuclear factor κB (NF-κB) signaling (11–14).

### 2.3 Trp metabolites: Aryl hydrocarbon receptor signaling networks

Trp metabolism by gut microbiota generates biologically active derivatives including indole, kynurenine (Kyn), and quinolinic acid, which act as key ligands for Aryl hydrocarbon receptor (AhR)—a ligand-dependent transcription factor critical for immune homeostasis (15, 16). Dysregulation of Trp metabolism contributes to inflammatory bowel disease, neuropsychiatric disorders, and cancer through mechanisms involving immune cell polarization and cytokine network modulation (15, 17). These metabolites exhibit pathway-specific effects: indole derivatives primarily activate AhR in epithelial and immune cells, while Kyn acts as both an AhR agonist and a metabolic checkpoint regulator (18). Trp metabolites form a complex signaling network centered on AhR, with divergent effects on T cell function determined by metabolite structure, concentration, and competing ligand availability in the TME (19).

### 2.4 Bile acids: nuclear receptor and GPCR crosstalk

Bile acids comprise hepatocyte-derived primary bile acids (cholic acid [CA], chenodeoxycholic acid [CDCA]) and microbiota-modified secondary bile acids (deoxycholic acid [DCA], lithocholic acid [LCA], ursodeoxycholic acid [UDCA]) (20). Their metabolism involves enterohepatic circulation with microbiota-mediated dehydroxylation and conjugation, generating ligands for both nuclear receptors and GPCRs (20, 21). The balance between primary and secondary bile acids, regulated by microbial enzymes like bile salt hydrolases, dictates overall immune tone in the TME (22). Bile acids function as metabolic messengers linking liver-gut axis homeostasis to tumor immunity, with secondary bile acids often exerting immunosuppressive effects in advanced malignancies (22).

### 2.5 Polyamines: metabolic reprogramming of immune cells

Polyamines are synthesized by gut microbiota including *Enterobacteriaceae* and *Bacteroides* species through arginine and ornithine decarboxylation (23). These metabolites regulate cellular proliferation and differentiation by modulating mRNA translation and autophagy, with context-dependent effects on tumor immunity (23, 24). Polyamines represent a double-edged sword in tumor immunity, promoting regulatory T cell (Treg)-mediated immunosuppression within the TME while supporting memory T cell development in secondary lymphoid organs, necessitating targeted delivery strategies.

### 2.6 Polysaccharides: pattern recognition receptor activation

Microbial polysaccharides, including extracellular polysaccharides (EPS) and capsular polysaccharides (CPS), exhibit structural heterogeneity that determines their interaction with pattern recognition receptors (PRRs) such as Toll-like receptors (TLRs) and C-type lectin receptors (25). These polysaccharides function as PRR agonists that bridge innate and adaptive immunity, with their structural diversity enabling targeted modulation of macrophage polarization and antigen-presenting capacity of dendritic cells (DCs) in the TME (26, 27).

### 2.7 LPS: TLR4-mediated inflammatory balance

LPS, a component of Gram-negative bacterial outer membranes, activates TLR4/MyD88 signaling to trigger inflammatory responses (28, 29). Structural variations in LPS, particularly lipid A acylation patterns, determine its potency and may explain strain-specific effects on cytokine release (28, 29). LPS exhibits temporal and dose-dependent effects on tumor immunity, with therapeutic potential in combination with radiotherapy or checkpoint inhibitors when delivered in controlled, localized formulations to avoid systemic toxicity (29, 30).

### 2.8 Other metabolites: emerging immunomodulatory pathways

Beyond major classes, diverse microbial metabolites modulate tumor immunity through specialized mechanisms. Trimethylamine N-oxide (TMAO), derived from dietary choline metabolism, enhances CD8^+^ T cell cytotoxicity via protein kinase R-like endoplasmic reticulum kinase (PERK)-dependent pyroptosis, despite promoting metastasis in other contexts (31–35). Urolithins, produced from ellagic acid, induce mitophagy in tumor-associated macrophages (TAMs) via transcription factor EB (TFEB) activation while expanding CD8^+^ T memory stem cells through Pink1-mediated mitochondrial regulation (36–38). Inosine modulates adenosine A2a receptor signaling to enhance CD8^+^ T cell function in glucose-deprived TMEs, serving as an alternative energy source through ribose phosphorylation (39–41). Desaminotyrosine (DAT) amplifies type I interferon (IFN-I) signaling via signal transducer and activator of transcription (STAT)1-mediated interferon alpha/beta receptor 1 (IFNAR1) upregulation, enhancing T cell priming (42). L-arginine (L-Arg) fuels nitric oxide (NO) production and T cell polyamine biosynthesis, counteracting myeloid-derived suppressor cell (MDSC)-mediated immunosuppression in acetate-enriched TMEs (43–45). These metabolites highlight the expanding landscape of microbial mediators that fine-tune immune responses through metabolic-immune crosstalk.

### 2.9 Integrated signaling networks

Microbial metabolites converge on three core signaling axes that unify their immunomodulatory functions: (1) Epigenetic regulation: SCFAs, Trp metabolites, and certain bile acids modulate chromatin accessibility to control immune cell fate decisions; (2)Metabolic-immune crosstalk: Nutrient-sensing pathways including mechanistic target of rapamycin (mTOR) (L-Arg) and PERK (TMAO) link metabolic state to immune cell activation; (3) PRR signaling: Polysaccharides (TLR2/4), LPS (TLR4), and certain indoles (AhR) activate conserved PRR pathways that bridge microbial sensing to adaptive immunity. These integrated networks exhibit context-dependent plasticity, with metabolite combinations often producing synergistic or antagonistic effects that cannot be predicted from individual components. For example, SCFAs enhance AhR expression in T cells, potentiating their responsiveness to Trp metabolites. Conversely, bile acid-mediated farnesoid X receptor (FXR) activation can antagonize SCFA-induced GPR43 signaling in hepatocytes. Understanding these interaction networks is critical for developing rational combination strategies in immunotherapy.

## 3 Impact of microbial metabolites on the TME

### 3.1 Impact on tumor cell growth

Microbial metabolites exhibit complex, context-dependent regulation of tumor cell fate, with butyrate representing a prime example of this duality—its biological effects are strongly concentration-dependent, superimposed on host genetics and metabolic context. At high concentrations (>10 mM) in the colonic lumen, butyrate serves as the primary energy source for normal colonocytes, supporting epithelial homeostasis via mitochondrial β-oxidation (46). When accumulated in colorectal cancer (CRC) cells at >100 μM (a concentration driven by Warburg effect-impaired butyrate oxidation), it functions as a potent HDAC inhibitor: this activity suppresses cell proliferation, induces apoptosis, and drives downstream effects like Pyruvate Kinase M2 activation (47) and reactive oxygen species (ROS)-induced apoptosis (48), resolving its paradox by prioritizing tumor suppression in malignant cells. In contrast, low concentrations (0.5-1.53 mM) of butyrate exert pro-tumorigenic effects in premalignant/genetically susceptible contexts (49, 50). Notably, ≥10 mM butyrate (e.g., 10–100 mM sodium butyrate) loses this pro-tumor effect, failing to stimulate CRC cell proliferation (49, 50). Host genetics, microbial co-metabolites (e.g., acetate/propionate synergizing with 0.69 mM butyrate to enhance senescence), and immune modulation further refine these outcomes. Propionate, meanwhile, delays mitochondrial-mediated apoptosis by inducing autophagy (51). Urolithin A (UA) and its structural analogs reduce CRC resistance to 5-fluorouracil by modulating the forkhead box O3 (FOXO3)-forkhead box M1 (FOXM1) axis (52). The Trp metabolite trans-3-indoleacrylic acid (IDA) promotes CRC by inhibiting ferroptosis through the AhR-aldehyde dehydrogenase 1 family member A3 (ALDH1A3) axis (53, 54). Additionally, *Reuterin* from healthy microbiota inhibits CRC growth by suppressing ribosome biogenesis through oxidative stress (55), while TMAO promotes CRC by inhibiting the FXR-fibroblast growth factor 15 (FGF15) axis and activating the Wnt/β-catenin pathway (56). Bile acids activate FXR and G protein-coupled bile acid receptor 5 (TGR5), triggering the mitogen-activated protein kinase (MAPK)/extracellular signal-regulated kinase (ERK) and phosphatidylinositol-3-kinase (PI3K)/protein kinase B (AKT) signaling cascades to drive tumor proliferation and anti-apoptosis (57–59). Research indicates that the gut microbiota can metabolize environmental carcinogens, thereby promoting the development of chemically induced tumors in distal organs and accelerating cancer progression (60). Cigarette smoke-induced dysbiosis elevates taurodeoxycholic acid (TDCA), activating MAPK/ERK, interleukin (IL)-17, and tumor necrosis factor (TNF) pathways to accelerate CRC (61). High-fat diet-associated lysophosphatidic acid directly stimulates cancer cell proliferation (62). The metabolite tyrosol inhibits CRC progression by suppressing NF-κB/hypoxia-inducible factor 1 (HIF-1) signaling, reducing ROS and inflammation (63), while indole imine and colibactin exacerbate CRC development through DNA damage (64, 65). Oncomicrobial LPS exhibits tissue-specific carcinogenicity through TLR4/C-C motif chemokine ligand (CCL)2 axis in esophageal cancer and S100A7/TLR4/receptor for advanced glycation end-products (RAGE) axis in breast cancer (66, 67). *Fusobacterium nucleatum*-derived ADP-heptose activates alpha kinase 1 (ALPK1)/TIFA axis, conferring CRC proliferation and multidrug resistance (68).

### 3.2 Regulation of tumor-associated immune cells

Microbial metabolites modulate tumor-associated immune cell dynamics through context-dependent, mechanistically distinct pathways, with SCFAs emerging as prime examples of concentration- and cell type-specific functional duality. At physiological concentrations (e.g., serum butyrate: ~2-5 μM in oxaliplatin responders, 69), SCFAs boost cytotoxic immunity via HDAC inhibition: 2 μM butyrate enhances natural killer (NK) cell cytotoxicity against myeloma by inducing extracellular vesicles and reducing IL-10 (69); 1–2 mM butyrate/10 μM acetate potentiates CD8^+^ T cells—butyrate drives inhibitor of DNA binding 2 (ID2)-dependent IL-12 signaling to upregulate IFN-γ/granzyme B (70); acetate shifts TAMs to M1 via Acetyl-CoA Carboxylase 1-mediated fatty acid biosynthesis (71, 72). SCFAs also shape mucosal immunity via GPR41 in CD4^+^ T cells, promoting AhR/HIF-1α-dependent IL-22 (73), while 500 μM-1 mM butyrate suppresses macrophage pro-inflammatory activation (74). Notably, SCFAs exhibit duality: 300 mM butyrate (murine drinking water) promotes colonic Treg differentiation via Foxp3 acetylation (75), yet intratumoral butyrate (>1 μM) inhibits DC function/IFN-I production to undermine radiotherapy (76)—highlighting the need for targeted delivery, supported by human data linking higher fecal/serum SCFAs to better therapy responses (70, 72). Beyond SCFAs, arginine reinforces CD8^+^ T cell activity and inhibits Tregs via mTOR signaling (77), whereas Trp metabolites exhibit divergent effects: Kyn induces CD8^+^ T cell exhaustion through AhR-dependent programmed death-1 (PD-1) upregulation (78), contrasting with DAT’s enhancement of IFN-I-primed T cell expansion (42). Bile acids modulate liver immunity by recruiting natural killer T (NKT) cells via the CXCL16-CXCR6 axis (79), and LPS exhibits dose-dependent immunomodulation, acutely activating T cells before promoting exhaustion during chronic exposure (80).

### 3.3 Remodeling the TME

Microbial metabolites orchestrate TME reprogramming through metabolic, epigenetic, and immune-stromal crosstalk, though their roles exhibit context-dependent duality requiring mechanistic prioritization. Immunosuppressive axes prominently feature: spermidine-driven suppression of CD8^+^ T cell function and Treg expansion (81), AhR-activated Kyn reinforcing Treg-macrophage inhibitory networks (82), and LPS/TLR4-mediated secretion of T cell/NK-suppressive factors (83)—the latter exhibiting strain-specific effects on cytokine release (84). Conversely, TME-sensitizing metabolites demonstrate therapeutic promise: TMAO enhances CD8^+^ T cell/M1 macrophage infiltration and IFN-γ/TNF-α production (85), while methylglyoxal synergizes with radiotherapy to induce immunogenic cell death (ICD) and cyclic guanosine monophosphate AMP synthase (cGAS)-stimulator of interferon genes (STING)-programmed death-ligand 1 (PD-L1) activation (86). Bacterial capsular polysaccharides (CHPS) polarize M1 macrophages via TLR2, triggering iron sequestration to starve tumors (87). Metabolic-stromal hijacking further shapes progression: agmatine stabilizes β-catenin via Rnf128 inhibition, activating Wnt-driven tumorigenesis (88); DCA induces epithelial-mesenchymal transition (EMT) and vasculogenic mimicry through vascular endothelial growth factor receptor 2 (VEGFR2) signaling (89), while in obesity-associated liver cancer, DCA triggers senescent hepatic stellate cells (HSCs) to secrete tumor-promoting factors (90). Crucially, even metabolites with dual roles demand context: SCFAs induce protumor autophagy/chemokine signaling in prostate cancer (91), yet propionylcarnitine exhibits antitumor effects by suppressing Tregs and key chemokines such as CCL20 and CXCL8 (92).

The TME-modulating effects of microbial metabolites are unified by three core principles: (1) concentration-dependent signaling (quantitative thresholds in human tissues define function), (2) cell-type/tissue specificity (e.g., butyrate’s intratumoral vs. systemic effects), and (3) cross-talk with host factors (diet, antibiotics [ATBs], genetics). Common mechanisms (HDAC inhibition, AhR activation, TLR signaling) integrate metabolite-specific effects, resolving the “patchwork” criticism. Translational progress (targeted delivery, engineered microbes) and cross-model validation (mouse vs. human data) provide a robust framework for developing metabolite-based TME reprogramming strategies.

## 4 The role of microbial metabolites in immunotherapy

### 4.1 The mechanisms of immunotherapy

Immunotherapy relies on three primary modalities: ICIs, adoptive cell transfer (ACT), and cancer vaccines, each harnessing unique immunological mechanisms to eliminate malignancies. ICIs antagonize immune checkpoint molecules (e.g., PD-1/PD-L1, cytotoxic T lymphocyte antigen-4 [CTLA-4]) to disinhibit cytotoxic T cell activity, reinvigorating antitumor immunity (93). Checkpoint proteins physiologically constrain T cell activation to prevent autoimmunity, but tumors co-opt this mechanism to evade immune destruction (93). ICI-mediated checkpoint blockade releases T cell effector functions, enabling tumor antigen recognition, clonal expansion, and target cell lysis (93). ACT entails ex vivo engineering of autologous or allogeneic T cells to express tumor-targeting receptors, followed by lymphodepletion and reinfusion to achieve sustained tumor control (94, 95). Chimeric antigen receptor T cell (CAR-T) therapy, a transformative ACT approach, genetically arms T cells with synthetic receptors (CARs) that redirect specificity toward tumor-associated antigens (96). Upon reinfusion, CAR-T cells engage tumor surface antigens, triggering perforin/granzyme-mediated apoptosis and pro-inflammatory cytokine storms (e.g., IFN-γ, IL-2) that amplify bystander immune activation (96). Cancer vaccines deliver tumor-associated antigens (e.g., neoantigens, shared antigens) via nanoparticle carriers or viral vectors to prime DC maturation, eliciting antigen-specific T cell responses (97, 98). Vaccines induce immunological memory through long-lived memory T cells and plasma cells, providing durable protection against tumor recurrence (97, 98). Clinical trials demonstrate that immunotherapy significantly enhances survival in specific cancer subtypes (99). However, approximately 70% of patients exhibit primary or acquired resistance (99), with therapeutic efficacy modulated by TME composition, immune cell infiltration dynamics, and metabolic reprogramming (100, 101). Deciphering these determinants is critical for optimizing therapeutic strategies and overcoming resistance.

### 4.2 The role of microbial metabolites in enhancing the efficacy of immunotherapy

**Figure 1:**
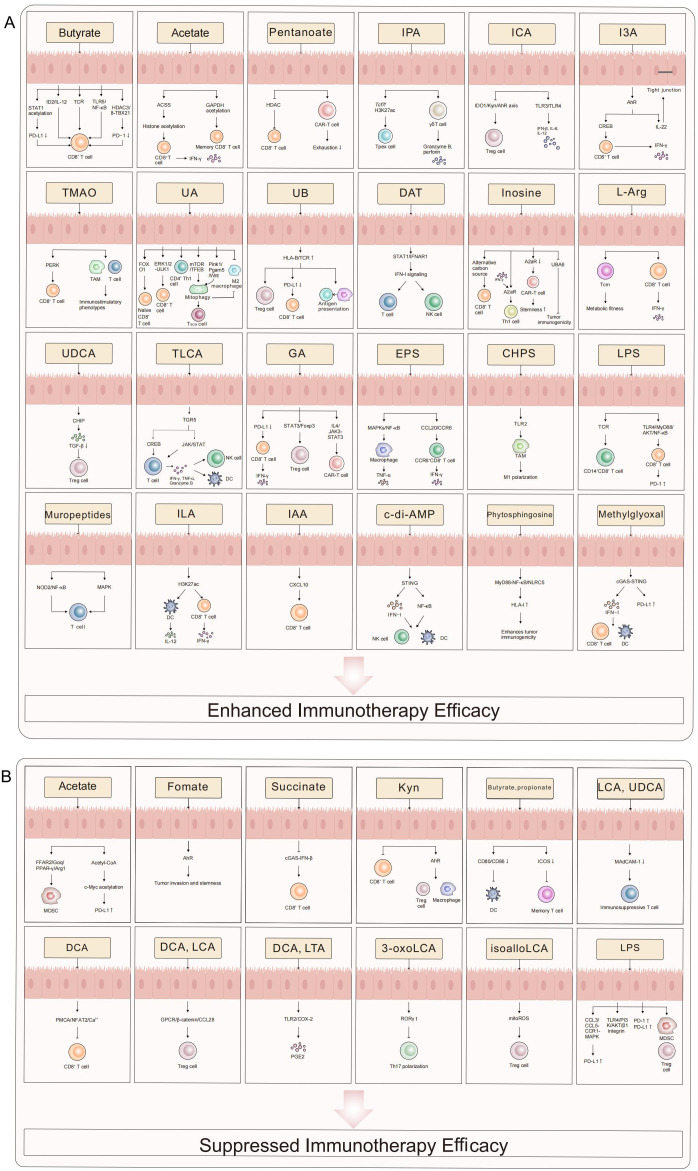
The role of microbial metabolites in tumor immunotherapy. This schematic systematically illustrates the bidirectional regulatory mechanisms of microbial metabolites in modulating immunotherapy efficacy. Enhancing Effects **(A)** SCFAs (e.g., butyrate, pentanoate) amplify CD8^+^ T cell cytotoxicity, memory differentiation, and TIL function via epigenetic reprogramming (STAT1 acetylation/HDAC inhibition), metabolic remodeling (ACSS-dependent histone acetylation), and immune checkpoint modulation (PD-L1/PD-1 downregulation). Indole derivatives (e.g., IPA, I3A) activate γδ T cell and Tc1 effector functions through AhR/CREB signaling while suppressing the IDO1/Kyn/AhR axis to attenuate Treg-mediated immunosuppression. Urolithins (UA/UB) and inosine sustain T cell stemness via mitophagy (Pink1/Pgam5), STING/IFN-I pathways, and metabolic adaptability. Suppressive Effects **(B)** Certain metabolites (e.g., succinate, secondary bile acids) foster an immunosuppressive microenvironment by activating AhR, upregulating PD-L1, or expanding MDSCs/Tregs. LPS and formate exacerbate T cell exhaustion through inhibitory signaling (TLR4/PI3K-AKT, cGAS-IFN-β suppression). This diagram delineates a molecular framework wherein microbial metabolic networks dynamically regulate antitumor immunity via “metabolic-epigenetic-immune” crosstalk, providing a theoretical basis for precision therapies targeting microbiota-host interactions. This figure was drawn using Adobe Illustrator software (https://www.adobe.com).

Microbial metabolites are pivotal mediators that translate gut microbiota composition into functional antitumor immunity. Their ability to enhance immunotherapy is not merely a collection of isolated effects but operates through a convergence on core immunologic pathways: epigenetic remodeling, metabolic reprogramming, and specific receptor signaling (e.g., AhR, GPCRs). This concerted action promotes cytotoxic T cell function, dampens immunosuppressive networks (Tregs, MDSCs), and reshapes the TME. However, the net effect of any single metabolite is profoundly context-dependent, governed by its concentration gradient, spatial distribution within the TME, host genetics, and the constellation of other present signals. The following sections detail key metabolites, emphasizing how their mechanisms exemplify these unifying principles while highlighting the specific challenges and opportunities they present for clinical translation. The collective evidence is summarized in **Table 1** and **Figure 1A**.

**Table 1:** Role of microbial metabolites in enhancing the efficacy of immunotherapy.

Microbial metabolite	Tumor type	Mechanism of action	Affected cells/targets	Related pathways/molecules	Synergistic immunotherapy	Refs.
Butyrate	CRC	Promotes STAT1 acetylation to inhibit PD-L1 expression, enhancing CD8^+^T cell cytotoxicity and infiltration.	CD8^+^T cells	STAT1	PD-1/PD-L1 inhibitor	(102)
Butyrate	CRC	Directly regulates anti-tumor CD8^+^ T cell responses via ID2-dependent IL-12 signaling pathway.	CD8^+^ T cells	ID2/IL-12	PD-1 inhibitor	(70)
Butyrate	NSCLC	Alters TCR signaling in cytotoxic CD8^+^ T cells, enhancing IFN-γ and TNFα synthesis.	CD8^+^ T cells, Vγ9Vδ2 T cells	TCR	PD-1 inhibitor	(103)
Butyrate	CRC	Activates CD8^+^ T cells via TLR5/NF-κB signaling pathway.	CD8^+^ T cells	TLR5/NF-κB	PD-1 inhibitor	(104)
Butyrate	MSS CRC	Downregulates PD-1 on CD8^+^ TILs via HDAC3/8-TBX21 axis, alleviating T cell exhaustion.	CD8^+^ TILs	HDAC3/8-TBX21	PD-1 inhibitor	(105)
Acetate	–	Restores IFN-γ production in glucose-restricted TILs via ACSS-dependent histone acetylation.	CD8^+^TILs	ACSS	–	(106)
Acetate	–	Enhances memory CD8^+^ T cell recall via GAPDH acetylation and glycolytic flux.	Memory CD8^+^ T cells	GAPDH	–	(107)
Pentanoate	Pancreatic Cancer	Inhibits HDAC class I enzymes to boost antigen-specific CD8^+^ T cell responses.	CD8^+^ T cells	HDAC/mTOR	CAR-T	(108)
Pentanoate	–	Pentanoate-engineered CAR-T cells reduce exhaustion and increase tumor infiltration.	CAR-T cells	–	CAR-T	(109)
IPA	Breast Cancer, Melanoma, CRC	Promotes Tpex cell generation via H3K27 acetylation in *Tcf7* super-enhancer regions.	Tpex	*Tcf7*/H3K27ac	PD-1 inhibitor	(110)
IPA	–	Stimulates γδT cells to secrete granzyme B and perforin, enhancing tumor killing.	γδT cells	–	–	(111)
ICA	CRC	Competitively antagonizes AhR binding by Kyn; suppresses IDO1 expression and Treg differentiation.	CD8^+^ T cells, Treg cells	IDO1/Kyn/AhR, TLR3/4	PD-1 inhibitor	(112)
I3A	Melanoma	Activates AhR/CREB signaling to drive CD8^+^ T cell IFN-γ production and cytotoxicity.	CD8^+^ T cells	AhR/CREB	PD-L1 inhibitor	(113)
I3A	–	Activates AhR/IL-22 to maintain gut barrier integrity, reducing ICIs toxicity.	Gut barrier cells	AhR/IL-22	ICIs	(114)
TMAO	TNBC	Induces tumor pyroptosis via PERK activation, boosting CD8^+^ T cell-mediated immunity.	CD8^+^ T cells, tumor cells	PERK	PD-1 inhibitor	(85)
TMAO	PDAC	Promotes immunostimulatory phenotypes in TAMs and T cells.	TAMs, T cells	–	PD-1 inhibitor	(34)
UA	–	Activates FOXO1 to promote naïve CD8^+^ T cell expansion and memory formation.	Naïve CD8^+^ T cells	FOXO1	–	(115)
UA	–	Induces TFEB-mediated mitophagy, reducing pro-inflammatory cytokines from macrophages.	TAMs	mTOR/TFEB	–	(116)
UA	–	Drives T_SCM_ cell formation via Pink1-dependent mitophagy.	CD8^+^ T cells	Pink1/Pgam5/Wnt	CAR-T	(37)
UA	PDAC	Reduces M2 macrophages, increases memory-like T cell infiltration and reduce interstitial fibrosis.	CD4^+^ Th1 cells, CD8^+^ T cells, M2 macrophages	–	PD-1 inhibitor	(117)
UA	–	Enhances CD8^+^ T cell metabolism via ERK1/2-ULK1 axis.	CD8^+^ T cells	ERK1/2-ULK1	–	(118)
UB	Colon Cancer	Suppresses Treg activity and PD-L1 expression, upregulates HLA-B/TCR and enhances antigen presentation.	Treg cells, CD8^+^ T cells	HLA-B/TCR	PD-1 inhibitor	(38)
DAT	–	Amplifies IFN-I signaling via STAT1/IFNAR1, activating T/NK cells.	CD4^+^ T cells, CD8^+^ T cells, NK cells	STAT1/IFNAR1	PD-1/CTLA-4 inhibitors	(42)
Inosine	–	Serves as alternative carbon source for T cell metabolism under glucose limitation.	CD8^+^ T cells	Purine metabolism	PD-L1 inhibitor, ACT	(119)
Inosine	CRC, Bladder Cancer, Melanoma	Drives Th1 differentiation via A2aR signaling in presence of IFN-γ.	CD4^+^ Th1 cells, CD8^+^ T cells	A2aR	CTLA-4/PD-L1 inhibitors	(41)
Inosine	Pan-cancer	Reprograms CAR-T metabolism via A2aR downregulation, enhancing stemness.	CAR-T cells	A2aR	CAR-T	(120)
Inosine	Pan-cancer	Inhibits UBA6 to enhance tumor immunogenicity and overcome ICIs resistance.	Tumor cells, CD8^+^ T cells	UBA6	ICIs	(121)
Inosine	Advanced Solid Tumors	Combined with PD-1/PD-L1 inhibitors delays progression and reduces toxicity.	–	–	PD-1/PD-L1 inhibitors	(122)
L-Arg	–	Regulates Tcm metabolic fitness and survival.	Tcm	–	–	(123)
L-Arg	Osteosarcoma	Boosts CD8^+^ T cell numbers and infiltration and increase the level of serum IFN-γ.	CD8^+^ T cells	–	PD-L1 inhibitor	(124)
L-Arg	Pan-cancer	Reverses acetate/FFAR2-mediated immunosuppression to overcome ICIs resistance.	CD8^+^ T cells	–	ICIs	(125)
Secondary Bile Acids	Melanoma	FMT elevates secondary bile acids to reverse PD-1 resistance.	–	–	PD-1 inhibitor	(126)
UDCA	Pan-cancer	Degrades TGF-β via CHIP-mediated autophagy, inhibiting Treg activation.	Treg cells	CHIP/TGF-β	PD-1 inhibitor	(127)
TLCA	NSCLC	Acts on T cells via the TGR5 receptor and JAK/STAT pathway, driving T cell activation and the production of effector molecules.	T cells	TGR5, JAK/STAT	ICIs	(128)
GA	NSCLC	Downregulates PD-L1, enhances PD-1-mediated tumor killing and increases IFN-γ secretion.	CD8^+^ T cells, tumor cells, PBMCs	–	PD-1 inhibitor	(129)
GA	CRC	Destabilizes STAT3/Foxp3 to inhibit Treg function and promotes IFN-γ production.	Treg cells, CD8^+^ T cells	STAT3	PD-1 inhibitor	(130)
GA	Lymphoma	Activates IL4/JAK3-STAT3 to enhance CD19 CAR-T efficacy.	CAR-T cells	IL4/JAK3-STAT3	CAR-T	(131)
EPS	–	Activates macrophages via MAPKs/NF-κB to secrete TNF-α.	Macrophages	MAPKs/NF-κB	–	(132)
EPS	CCL20^+^ Tumors	Recruits CCR6^+^CD8^+^ T cells to produce IFN-γ.	CCR6^+^CD8^+^ T cells	CCL20/CCR6	CTLA-4/PD-1 inhibitors	(27)
CHPS	–	Activates TAM polarization to M1 phenotype via TLR2; induces CD8^+^ T cell response; promotes tumor apoptosis via iron deprivation.	TAMs; CD8^+^ T cells	TLR2	PD-L1 inhibitor	(87)
LPS	–	Enhances anti-tumor effector function of CD14^+^CD8^+^ T cells.	CD14^+^CD8^+^ T cells	TCR	–	(133)
LPS	PDAC	Upregulates PD-L1 via TLR4/MyD88/AKT/NF-κB but synergizes with PD-L1 blockade.	CD3^+^ T cells, CD8^+^ T cells	TLR4/MyD88/AKT/NF-κB	PD-L1 inhibitor	(80)
LPS	Pan-cancer	Combined with PGA reshapes TME and gut microbiota.	CD8^+^ T cells, Tregs	–	PD-L1 inhibitor	(134)
Muropeptid-es	Pan-cancer	Activates NOD2/NF-κB and MAPK pathways to boost PD-L1 therapy.	T cells	NOD2/NF-κB, MAPK	PD-L1 inhibitor	(135)
ILA	–	Epigenetically enhances IL-12 production in DCs via H3K27ac modification; promotes CD8^+^ T cell priming.	DCs, CD8^+^ T cells	H3K27ac	–	(136)
IAA	–	Promotes CXCL10-mediated CD8^+^ T cell recruitment.	CD8^+^ T cells	CXCL10	PD-1 inhibitor	(137)
C-di-AMP	Pan-cancer	Activates STING pathway in APCs; induces IFN-I and enhances NK-DC crosstalk.	Monocytes, NK cells, DCs	STING	PD-1/PD-L1 inhibitors	(138)
Phytosphin-gosine	–	Upregulates HLA class I via MyD88–NF-κB/NLRC5 axis; enhances tumor immunogenicity	Tumor cells	MyD88/NF-κB/ NLRC5, HLA-I	ICIs	(139)
Methylglyo-xal	CRC	Enhances endoplasmic reticulum stress and ICD; activates cGAS-STING pathway; upregulates PD-L1 expression.	Tumor cells, DCs	cGAS-STING; PD-L1	PD-1 inhibitor combined with radiotherapy	(86)
Isobutyric acid	CRC	Increases CD3^+^ T cell infiltration via G protein-coupled receptor activation and histone modification.	CD3^+^ T cells	G protein-coupled receptor; histone	PD-1 inhibitor	(140)
Trigonelline	Bladder cancer	Suppresses β-catenin expression to promote CD8^+^ T cell infiltration.	CD8^+^ T cells	β-catenin	PD-1 inhibitor	(141)
DHA	Melanoma	Binds PD-L1 promoter to inhibit c-Myc-mediated PD-L1 transcription.	Tumor cells	c-Myc/PD-L1 transcription	PD-L1 inhibitor	(142)
DHA	–	Induces PD-L1 protein destabilization via ubiquitin-proteasomal and CSN5-mediated lysosomal degradation.	PD-L1 protein	Ubiquitin-proteasome; CSN5	PD-L1 inhibitor	(143)
DHA	NSCLC	Alters membrane phospholipid composition to reduce PD-1/PD-L1 binding; increases TIL infiltration and activation.	TILs	Membrane phospholipid	PD-1/PD-L1 inhibitors	(144)
Mevalonate	NSCLC	Stabilizes *CD274* mRNA to upregulate PD-L1 expression; enhances cytotoxic T cell function.	CD3^+^ T cells, CD8^+^ T cells	*CD274* mRNA	PD-L1 inhibitor	(145)
Ascorbic acid	–	Expands CD11b^+^CD44^+^PD-L1^+^ cell population; inhibits SLC7A11/GPX4-mediated ferroptosis.	CD11b^+^CD44^+^PD-L1^+^ cells	SLC7A11/GPX4	PD-1 inhibitor	(146)
Tetrahydro-biopterin	PDAC	Restores tetrahydrobiopterin/dihydrobiopterin ratio to reduce MDSC recruitment.	MDSCs	Biopterin metabolism	ICIs	(147)

#### 4.2.1 Butyrate: a paradigm of concentration-dependent epigenetic immunomodulation via the “concentration-cell-therapy” axis

Butyrate, a key gut microbial SCFA, embodies a unified “concentration-cell type-therapy context” framework that resolves its dual immunomodulatory effects, linking epigenetic regulation to therapeutic outcomes. Quantitatively, its functional thresholds align with prior observations: gut lumen physiological concentrations reach 10–20 mM (105), while therapeutic tumor levels require targeted delivery to 1–5 mM (70, 102) (systemic concentrations >0.5 mM risk immunosuppression; 76). Clinically, this translates to prognostic value: serum butyrate ≥0.3 μM correlates with oxaliplatin response (vs. <0.1 μM in non-responders) via enhanced human CD8^+^ T cell ID2/IFN-γ (70), and fecal butyrate ≥40 μmol/g associates with 2.3-fold higher anti-PD-1 progression-free survival (PFS) in solid tumor patients (148).

Mechanistically, butyrate acts via class I HDAC inhibition—an epigenetic hub unifying its effects. In CD8^+^ T cells, 1–5 mM butyrate increases H3K9/14 acetylation at *Id2*/*Tbx21* promoters (70, 149), driving ID2-dependent IL-12R upregulation and boosting IFN-γ/granzyme B production (70), as previously observed. In human CRC, it acetylates STAT1 to block PD-L1 transcription, restoring CD8^+^ T cell cytotoxicity (103, 104). It also enhances gut barrier function via claudin-3 upregulation (105), reducing microbial translocation and immune-related adverse events (irAEs) (150, 151).

This axis is context-dependent: systemic concentrations >1 mM promote Foxp3^+^ Treg differentiation via *Foxp3* acetylation (75)—increasing colonic Tregs, potentially driving anti-CTLA-4 resistance (152). It also suppresses DCs: 2 mM butyrate blocks STING-dependent phosphorylation, reducing IFN-β by 50% and undermining radiotherapy (76), as noted earlier.

Translational challenges are addressable via precision strategies: high-fiber diets increase fecal butyrate by 2.1-fold (148), while vancomycin reduces serum butyrate by 60% (76), necessitating patient stratification. Delivery approaches include engineered *Roseburia intestinalis* (boosting tumor butyrate to 2–3 mM in CT26 models; 105), enteric-coated butyrate (3-fold gut bioavailability; 69), and TME-responsive nanoparticles (2.5-fold CD8^+^ T cell infiltration in MC38 tumors; 105).

In summary, butyrate’s utility depends on aligning concentration with therapy needs: localized 1–5 mM activates CD8^+^ T cells/anti-PD-1 efficacy, while systemic >0.8 μM risks Treg-driven immunosuppression. Resolving cancer-specific thresholds and validating delivery strategies in phase II trials will realize its potential, emphasizing precision targeting for microbial metabolites.

#### 4.2.2 Acetate: a metabolic-epigenetic integrator of T cell function via context-dependent acetyl-CoA signaling

Acetate acts as a pivotal microbial metabolite unifying T cell metabolic adaptation and epigenetic regulation, operating through a context-dependent “acetyl-CoA pool dynamics” framework—its effects are dictated by acetyl-CoA synthetases (ACSS1/2)-mediated conversion to acetyl-CoA, which modulates glyceraldehyde-3-phosphate dehydrogenase (GAPDH) and histone acetylation to govern T cell metabolism, survival, and effector function (106, 107, 153). Quantitative thresholds define its activity: in murine L6 myotube cells, 0.5 mM acetate induces peak GPR43-dependent intracellular calcium influx (suppressed at >1 mM; 114); human CD4^+^ T cells require 10 mM acetate to augment GAPDH acetylation and glycolysis, driving Th1 polarization (106); systemic bacterial infection elevates serum acetate to 2–5 mM, optimizing memory CD8^+^ T cell recall responses (107). These contrast with homeostatic levels (0.1–1 mM in humans/mice), establishing “low” (≤1 mM) and “high” (≥2 mM) thresholds for context-dependent function.

Mechanistically, acetyl-CoA targets three key pathways in T cells. In effector Th1/CD8^+^ T cells, acetate-derived acetyl-CoA acetylates GAPDH to boost glycolysis, critical for IFN-γ production—conserved across species, as 5 mM acetate increases GAPDH acetylation by 2.3-fold and IFN-γ in human PD-1^+^ exhausted CD8^+^ T cells, while accelerating murine memory T cell glycolytic recall (107, 153). Under glucose deprivation, ACSS2-dependent acetyl-CoA restores H3K9/14 and H3K27 acetylation in exhausted CD8^+^ T cells, reactivating effector gene loci (Ifng, Tbx21) and chromatin accessibility—effects that are abrogated in ACSS2-deficient T cells (153). For T cell survival, acetate further acetylates α-tubulin to stabilize microtubules, a process antagonized by CD30 that physically associates with HDAC6 to deacetylate α-tubulin (154). This negative feedback loop—acetate upregulates CD30 transcription via H3K27 acetylation, while CD30 limits α-tubulin acetylation—prevents excessive T cell survival and maintains immune homeostasis (154).

Translational relevance is supported by cross-species data: 5 mM acetate enhances anti-CD3/CD28-induced IFN-γ by 40% in human effector memory CD8^+^ T cells, while acetate-augmented murine memory T cells reduce Listeria liver burden by 2.5-fold (107). Critical challenges include confounders (high-fiber diets increase fecal acetate by 2.1-fold; vancomycin cuts serum acetate by 60% in mice) and delivery—targeted strategies (ex vivo T cell ACSS2 upregulation, pH-sensitive nanoparticles for tumor-specific release) are essential to avoid off-target effects (e.g., tumor lipid biosynthesis; 112, 115). Systemic administration risks metabolic perturbations (e.g., diabetic insulin resistance), requiring dose trials to define safe windows (106, 155).

In summary, acetate’s utility lies in integrating T cell metabolism/epigenetics via acetyl-CoA pool dynamics. Realizing this potential requires aligning concentration with cell-type needs (2–5 mM for memory T cells, 10 mM for Th1 polarization), addressing confounders, and advancing targeted delivery—translating its context-dependent mechanisms to clinical benefit.

#### 4.2.3 Pentanoate: an emerging synergistic epigenetic adjuvant via metabolic-epigenetic crosstalk

Pentanoate (valerate), a microbial SCFA with unique immunomodulatory potential, distinguishes itself from other SCFAs by unifying metabolic reprogramming and epigenetic modulation—a conceptual framework that addresses the fragmentation of SCFA-mediated antitumor immunity and links microbial metabolism to T cell effector function (108, 109, 156). Its core mechanism involves selective inhibition of class I HDAC (HDAC1/2) to boost histone H3K9/14 acetylation in CD8^+^ T cells, while its 5-carbon structure enables dual entry into the tricarboxylic acid cycle (via acetyl-CoA and succinyl-CoA post β-oxidation). This drives citrate production, which adenosine triphosphate (ATP)-citrate lyase shuttles to the nucleus to sustain acetylation—reinforcing effector gene expression (IFN-γ, TNF-α, CD25) more effectively than standalone HDAC inhibitors like mocetinostat (109). Concurrently, it activates mTOR to enhance glycolysis and IL-2 autocrine signaling, synergizing with butyrate for robust antigen-specific T cell expansion (108).

This mechanism translates consistently across preclinical and clinical models: in immunocompetent mice, pentanoate-engineered CAR-T cells exhibit superior tumor control through increased infiltration, reduced exhaustion markers, and preferential differentiation toward naive-like phenotypes (109). Clinically, two independent cohorts confirm relevance: German CAR-T patients with high fecal pentanoate (≥74.1 μg/g) had a 1-year PFS of 90% vs. 41.5% in low-pentanoate groups, and U.S. patients in the top pentanoate tertile showed numerically longer 2-year PFS (109). Critically, confounding factors like ATB use (e.g., piperacillin/tazobactam, imipenem) deplete pentanoate-producing gut commensals (e.g., *Megasphaera massiliensis*) and reduce fecal pentanoate levels, correlating with worse CAR-T outcomes—underscoring the need for patient stratification by microbial status (109).

Notably, translational delivery is optimized by ex vivo pretreatment during CAR-T cell manufacturing (2-day exposure during activation) rather than systemic administration: *in vivo* pentanoate fails to enhance anti-PD-1 therapy, whereas ex vivo programming preserves its metabolic-epigenetic effects and avoids off-target immunosuppression (e.g., Treg induction, which is triggered by butyrate but not pentanoate) (108, 109). Despite these advances, challenges remain: tumor-intrinsic heterogeneity (e.g., variable ATP-citrate lyase expression) may limit efficacy in ATP-citrate lyase-low tumors, and strain-specific microbial engineering (to boost pentanoate production) requires refinement to avoid interpatient variability. Collectively, pentanoate exemplifies how microbial metabolites can bridge metabolic and epigenetic regulation to enhance cellular immunotherapy—offering a actionable, mechanism-driven adjuvant strategy that aligns preclinical mechanistic insights with clinical outcomes.

#### 4.2.4 Indole derivatives: fine-tuning immunity through the AhR axis

The immunomodulatory effects of tryptophan metabolites—Indole-3-propionic acid (IPA), Indole-3-carboxylic acid (ICA), and Indole-3-aldehyde (I3A)—collectively illustrate the principle of context-dependent AhR signaling. AhR activation is not intrinsically good or bad; its functional outcome is a product of the specific ligand, its concentration, and the cellular and tissue milieu.

IPA represents the best-characterized indole metabolite due to robust causal evidence. While exhibiting basal roles in glycemic/lipid regulation and gut barrier preservation (157), its bioavailability is dynamically governed by dietary Trp, microbiota composition, and host metabolism (158)—with causal validation showing that IPA cannot be produced by *Lactobacillus johnsonii* alone (which only generates indole-3-lactic acid [ILA]) but requires cooperation with *Clostridium* sp*orogenes* to metabolize ILA into IPA (110). This microbial synergy is indispensable: germ-free mice colonized with both strains, but not either alone, exhibit elevated plasma IPA and enhanced aPD-1 efficacy (110). Mechanistically, causal evidence from gain- and loss-of-function studies demonstrates IPA epigenetically sustains CD8^+^ T cell stemness via H3K27 hyperacetylation at the *Tcf7* super-enhancer region (110). This reprogramming drives progenitor-exhausted T cell (Tpex) differentiation, as shown by: (1) IPA supplementation in breast cancer, melanoma, and CRC models synergizing with PD-1 blockade to increase CD8^+^ tumor-infiltrating lymphocyte (TIL) frequency and T-cell factor 1 (TCF-1) expression; (2) *Tcf7* knockout abrogating IPA’s ability to enhance immunotherapy, confirming *Tcf7* as a critical mediator; (3) adoptive transfer of IPA-pretreated CD8^+^ T cells (but not untreated cells) restoring aPD-1 responsiveness in Rag1^-^/^-^ mice, with CD8 neutralization eliminating IPA’s efficacy (110). Pan-cancer studies further validate IPA’s causal role: its supplementation improves ICI response rates across malignancies (110, 159), while ATB-induced microbiota depletion reduces IPA levels and blunts γδ T cell-mediated tumor control—an effect reversed by exogenous IPA, which directly upregulates granzyme B/perforin in γδ T cells (111). Collectively, these experiments establish IPA as a microbial metabolite with causal roles in enhancing immunotherapy, warranting therapeutic prioritization. The requirement for microbial consortia highlights a major translational hurdle: bacterial teamwork is often essential for generating the most beneficial metabolites, complicating simple probiotic approaches.

ICA, a metabolite with dual origins—derived both from gut microbial Trp catabolism and the breakdown of compounds in cruciferous vegetables (112, 160–163)—exemplifies ligand-specific modulation of the AhR and competitive interplay within critical immunometabolic pathways. At physiological concentrations (e.g., approximately 5 μM in tumor tissues), ICA acts as a competitive partial AhR agonist, binding to AhR with higher affinity than the immunosuppressive ligand Kyn (112). This interaction not only antagonizes Kyn-induced AhR activation but also suppresses the expression of indoleamine 2,3-dioxygenase (IDO)1—a key enzyme driving Trp catabolism to Kyn—and subsequent differentiation of Tregs, thereby reversing the immunosuppressive TME (112, 161). This effect is tightly dependent on the tumor’s enzymatic landscape, particularly IDO1 expression levels, which explains ICA’s synergistic activity with anti-PD-1 therapy in both microsatellite instability-high (MSI-H) and microsatellite instability-low (MSI-L) CRC models (112); in these settings, ICA reduces intratumoral Treg infiltration and enhances CD8^+^ T cell cytotoxicity, even overcoming the inherent immunotherapy resistance of MSI-L tumors. In contrast, in macrophage-like cells, synthetic ICA derivatives (at concentrations of 10.8–21.6 μM) elicit a distinct response: within 4 hours of exposure, they activate endosomal TLR3 and surface TLR4 (164), triggering cascades of type I interferons (e.g., IFNB1) and proinflammatory cytokines (e.g., IL6, IL12A/B) that amplify innate antiviral immunity—underscoring ICA’s context-dependent functional plasticity. Despite this versatility, ICA’s pan-cancer potential is limited by AhR activation thresholds: in tumors with AhR overexpression (e.g., glioblastoma, pancreatic cancer), its weak agonistic activity may paradoxically sustain immunosuppression by reinforcing AhR-mediated pro-tumor signaling, rather than exerting antagonistic effects. Additionally, tumors with low IDO1 expression show reduced sensitivity to ICA’s modulation of the Kyn-AhR axis, as limited Kyn production diminishes the competitive advantage of ICA’s AhR binding. Collectively, ICA’s therapeutic efficacy hinges on dynamic host-microbiome co-metabolism, where tissue-specific enzymatic profiles (e.g., IDO1 abundance) and immune cell composition (e.g., Treg frequency, macrophage polarization) dictate its functional outcomes—highlighting the need for context-aware patient stratification to maximize its utility in immunotherapeutic strategies.

I3A, a microbial-host co-metabolite, exerts immunomodulatory effects unified by AhR-mediated concentration thresholds and tissue-specific spatial compartmentalization—a framework supported by both preclinical mechanistic validation and clinical correlative data. Derived from bacterial Trp catabolism (e.g., *Lactobacillus reuteri* via aromatic amino acid aminotransferase) and dietary indole-3-carbinol (I3C) (165, 166), I3A exerts context-dependent functions defined by quantifiable concentration ranges and AhR signaling specificity. At low concentrations (10–50 μM, physiological levels in the intestinal mucosa), I3A reinforces epithelial barrier integrity by triggering AhR-dependent transcription of IL-22 and suppressing NF-κB/IL-6 signaling (167–170). This mitigates inflammation-driven carcinogenesis by upregulating tight-junction proteins and inhibiting myosin light-chain kinase-mediated barrier disruption (168, 170). In colitis models, this concentration range reduces gut permeability and epithelial apoptosis (168). At therapeutic concentrations (100–200 μM, achievable via intratumoral delivery), I3A activates the AhR/cAMP response element-binding protein (CREB) axis in CD8^+^ T cells: specifically, AhR recruits CREB to the IFN-γ promoter, inducing CREB phosphorylation at Ser133 to amplify type 1 cytotoxic T cell (Tc1) differentiation and tumor cytotoxicity (113, 171). Loss-of-function studies validate this mechanism: *L. reuteri* ΔAAT (a Trp catabolism-deficient strain) fails to produce I3A or suppress melanoma growth, while CD8^+^ T cell-specific AhR knockout completely abrogates I3A-driven tumor suppression (113). Conversely, concentrations exceeding 300 μM (systemic overexposure) cause AhR hyperactivation, inducing Foxp3^+^ Tregs via AhR-dependent Foxp3 locus acetylation and upregulating IDO1 in DCs—narrowing the therapeutic window (114, 170). Preclinical models show that intratumoral I3A (150–200 μM) delivered via engineered probiotics (e.g., *L. reuteri* overexpressing Trp 2,3-dioxygenase) enhances anti-PD-L1 efficacy in melanoma (113). This aligns with clinical data: in advanced melanoma patients receiving anti-PD-1/IFNα, serum I3A levels >31.15 pmol/100 μL (70th percentile) correlate with significantly longer PFS (12.6 vs. 5.8 months) and OS (28.3 vs. 14.1 months), whereas non-responders show no differences in levels of the endogenous AhR ligand Kyn—confirming I3A-specific effects (113). Confounders including dietary Trp/I3C intake and host genetics are critically addressed: a Trp-rich diet potentiates I3A-mediated antitumor effects by increasing intratumoral I3A concentrations, but requires standardized dietary assessment in clinical trials (113). Host AhR polymorphisms diminish I3A-induced CD8^+^ T cell activation, highlighting the need for patient stratification by AhR genotype to improve predictive accuracy (113). Feasible translational delivery strategies include engineered probiotics (achieving 150–200 μM intratumoral I3A with serum levels <10 nM), enteric-coated I3A formulations (poly microspheres, increasing gut bioavailability by 3-fold vs. free I3A), and direct intratumoral injection (200 μg/mL I3A)—all of which maximize efficacy while avoiding systemic toxicity (113, 114, 170). In conclusion, the clinical translation of I3A relies on leveraging its AhR-dependent concentration thresholds (100–200 μM intratumoral, >31.15 pmol/100 μL serum) and tissue-specific signaling. Future studies should prioritize controlled dietary Trp interventions in ICI trials, Phase I/II trials of I3A-loaded engineered probiotics in melanoma, and validation of I3A as a predictive biomarker—positioning I3A as a prototype for microbiome metabolite-based adjuvants to optimize ICI therapy.

#### 4.2.5 TMAO: context-dependent inflammomodulation and the confounder challenge

TMAO powerfully demonstrates the cancer-type specificity of metabolite actions. While associated with progression in colorectal, liver, pancreatic, and breast cancers through mechanisms like endothelial NF-κB/vascular endothelial growth factor A (VEGFA)-driven angiogenesis and periostin (POSTN)-mediated Integrin-linked protein kinase (ILK)/AKT/mTOR activation that fuels EMT and metastasis in hepatocellular carcinoma (HCC) (172–176), TMAO simultaneously enhances ICI response via context-dependent immunostimulation—with causality established through multiple experimental approaches. In triple-negative breast cancer (TNBC), TMAO improves ICI response via PERK-dependent pyroptosis: gasdermin E knockout blocks pyroptosis and anti-PD-1 synergism; PERK inhibition reverses CD8^+^ T cell activation (85, 177). Clinically, high plasma TMAO correlates with enhanced CD8^+^ T cell cytotoxicity and prolonged PFS, with TMAO-treated CD8^+^ T cells showing stronger tumor-killing capacity *in vitro* (85). In pancreatic ductal adenocarcinoma (PDAC) models, microbiota depletion via metronidazole reduces serum TMAO 73-fold and increases tumor burden, reversed by TMAO/trimethylamine (TMA) supplementation; CutC/D inhibition lowers TMAO and exacerbates growth, while choline-rich diets (boosting TMAO) reduce tumors and induce immunostimulatory TAMs. Notably, depleting macrophages or CD8^+^ T cells abrogates TMAO’s efficacy, confirming their essential role (34, 178).

However, its translational validity is heavily challenged by significant confounders. Plasma TMAO levels are directly modulated by dietary choline/red meat intake (31), host flavin-containing monooxygenase enzyme activity, and renal function. This raises a pivotal question: is TMAO a causal therapeutic agent, or merely a biomarker of dietary intake and host metabolic capacity? Furthermore, its detrimental off-target effects—promoting insulin resistance, cardiovascular disease, and neurodegeneration through chronic PERK activation (179, 180)—necessitate extremely careful therapeutic window definition and sophisticated tumor-targeted delivery strategies (e.g., PERK-activating nanoparticles) to avoid unacceptable systemic toxicity.

#### 4.2.6 Urolithins: mitophagy-mediated enhancement of immune cell fitness

Urolithins enhance immunotherapy by orchestrating mitochondrial fitness and metabolic reprogramming across cell types. Urolithins represent a family of pleiotropic microbial metabolites (≥10 conjugated derivatives) with tumor-agnostic therapeutic potential, operating through dual tumor-intrinsic and immunomodulatory mechanisms. Beyond established anti-inflammatory and antioxidant properties (181, 182), urolithins directly suppress tumorigenesis via concerted modulation of key oncogenic pathways—including AKT/WNK1 (183), p53/mdm2/Snail (184), p53/TIGAR (185), Wnt/β-catenin (186), and PI3K/AKT/mTOR cascades (187, 188)—culminating in cell cycle arrest and apoptosis across malignancies (183–190).

Critically, their most promising translational value lies in TME reprogramming and immunotherapy potentiation: UA reshapes antitumor immunity through context-specific metabolic tuning—activating forkhead box O1 (FOXO1) to drive CD8^+^ T cell expansion and memory formation independent of mitophagy (115), while concurrently inducing TFEB-mediated mitophagy in TAMs to suppress IL-6/TNF-α and mitigate inflammation (116). In CD8^+^ T cells, UA triggers PTEN-induced kinase 1 (Pink1)-dependent mitophagy that releases mitochondrial phosphatase Pgam5, potentiating Wnt signaling to generate T memory stem cells (T_SCM_) and enhancing CAR-T_SCM_ expansion (37). This metabolic-immune crosstalk directly overcomes resistance mechanisms: in PDAC models, UA reduces stromal fibrosis, downregulates PD-1, and redirects macrophage polarization from M2 to immunostimulatory phenotypes, synergizing with anti-PD-1 therapy to enhance Th1-polarized T cell infiltration and improve survival (117). UA further sustains CD8^+^ T cell persistence via the ERK1/2-ULK1 axis, optimizing metabolic fitness and ROS homeostasis through autophagic flux (118).

Complementarily, urolithin B (UB) suppresses immunosuppressive networks by inhibiting Treg activity and PD-L1 expression while upregulating human leukocyte antigen (HLA)-B and T-cell receptor (TCR) molecules to enhance antigen presentation—effectively mimicking DC vaccines (38). UB simultaneously reprograms immunoregulatory gut microbiota in CRC, creating synergistic antitumor effects when combined with checkpoint blockade (38). Their pleiotropic effects position them as multi-mechanism agents, but their notoriously poor oral bioavailability remains a major barrier (181), demanding advanced formulation strategies (e.g., nanoparticles, phospholipid complexes) to achieve therapeutic efficacy.

#### 4.2.7 DAT: type I interferon amplification with limited clinical scope

IFN-I are key regulators of antitumor immunity, orchestrating DC cross-priming, CD8^+^ T cell activation, and NK cell cytotoxicity—core processes supporting ICI efficacy (191–193). The gut microbial metabolite DAT amplifies this pathway via a unified STAT1-IFNAR1 positive feedback loop: it enhances STAT1 phosphorylation to upregulate IFNAR1 expression, creating a cascade that potentiates IFN-I signaling (42, 194). This mechanism not only protects against viral infections (e.g., influenza, 164) but also boosts antitumor immunity by dual immunomodulation, enhancing IFN-I-driven CD8^+^ T cell priming (42, 195).

Preclinically, DAT’s efficacy relies on quantifiable concentration thresholds and intact gut microbiota: the therapeutic dose range (125–200 mg/kg/day, oral) aligns with its no-observed-adverse-effect level in rodents (196), driving tumor-specific immune activation (42) while preserving gut microbiota diversity (e.g., enriching beneficial *Burkholderiales*, 42). In contrast, doses >250 mg/kg induce dose-dependent multiorgan toxicity (elevated liver transaminases, renal dysfunction) and disrupt gut microbiota balance (e.g., reduced *Bacteroidales*, 167).

Translational challenges for DAT include model-specific efficacy differences: it enhances anti-CTLA-4 efficacy in B16-OVA melanoma (42) but fails to augment the abscopal effect in MC38 colon adenocarcinoma (197), attributed to MC38’s low immunogenicity and reduced IFN-I responsiveness—highlighting the need for clinical patient stratification by tumor IFN-I gene signatures (e.g., OAS2/Mx2 expression, 165). Feasible solutions include engineered probiotic vectors (e.g., *Flavonifractor plautii* overexpressing flavonoid-degrading enzymes) to achieve 150–200 μM intratumoral DAT with serum levels <10 nM (42), and enteric-coated poly (lactic-co-glycolic acid) microspheres that boost gut bioavailability by 3-fold vs. free DAT (196). Additionally, clinical trials must standardize dietary flavonoid intake and assess baseline microbiota (e.g., *F. plautii* abundance, a major DAT producer) to control variability, as DAT can reverse ATB-induced ICI resistance without disrupting beneficial taxa at therapeutic doses (42).

In summary, DAT’s translational potential lies in leveraging its STAT1-IFNAR1 regulatory axis within a defined therapeutic window (125–200 mg/kg/day oral, 150–200 μM intratumoral). Future work should prioritize Phase I trials of probiotic-delivered DAT in melanoma (stratified by IFN-I signature), human intestinal organoid models for toxicity validation, and controlled dietary interventions—unifying its mechanistic, preclinical, and translational properties.

#### 4.2.8 Inosine: a metabolic fuel with a dichotomous signaling profile

Inosine functions as a unifying immunometabolite that integrates immune signaling modulation, metabolic reprogramming, and tumor immunogenicity enhancement—three interconnected mechanisms rather than isolated effects—with its anticancer activity centered on rewiring the tumor immune microenvironment in a context-dependent manner (119, 121, 198, 199). Beyond its established roles in infections and inflammation (198), its efficacy relies on defined concentration thresholds, tumor-intrinsic factors (e.g., ubiquitin-like modifier activating enzyme 6 [UBA6] expression), and TME nutrient status.

Mechanistically, inosine operates through three core linked processes: First, as an alternative carbon source for nutrient-deprived effector T cells, it is hydrolyzed by purine nucleoside phosphorylase to ribose-1-phosphate, which fuels glycolysis and the pentose phosphate pathway to sustain ATP production (119). Intratumoral concentrations of 100–200 μM maintain CD8^+^ T cell proliferation and IFN-γ secretion under glucose restriction (vs. <50 μM failing to reverse exhaustion), validated in B16 melanoma mice where inosine boosts intratumoral CD8^+^ T cells by 2.3-fold (119). Second, it drives M1 macrophage polarization to suppress CRC: In CT26 tumor-bearing mice, intraperitoneal inosine (5–50 mg/kg/day) dose-dependently upregulates M1 markers and reduces M2 markers, with 50 mg/kg achieving 47.39% tumor inhibition via NF-κB/IL-1β activation (199). Third, it enhances tumor immunogenicity by binding and inhibiting UBA6 in tumor cells to elevate immunogenicity and overcome therapeutic resistance (121).

A key contextual duality arises from adenosine A2a receptor (A2aR) signaling: Inosine promotes Th1 differentiation when IFN-γ is abundant (10–20 ng/mL in inflamed TIME) via A2aR/cAMP/CREB phosphorylation, but inhibits Th1 in IFN-γ-deficient contexts (198, 200). DC-derived IL-12 is critical to resolve this duality, as DC depletion abrogates inosine’s Th1 effect (41). Preclinically, 100 μM inosine preconditioning enhances CAR-T cell efficacy by increasing T_SCM_ frequency and achieving complete tumor regression in 50% of 4T1 breast cancer mice (vs. 0% in untreated CAR-T groups) (120, 201).

Translational challenges are addressed by evidence-based solutions: A therapeutic window (50–200 mg/kg/day in mice) preserves gut microbiota and avoids toxicity (doses >250 mg/kg induce liver/kidney damage and dysbiosis) (119); in humans, serum inosine >31.15 pmol/100 μL correlates with improved anti-PD1 response in melanoma (198). Feasible delivery includes engineered probiotics, enteric-coated poly (lactic-co-glycolic acid) microspheres (119, 199), and intratumoral injection of 200 μg/mL inosine; the derivative isoprinosine (oral 500 mg tid) is safe in phase 4 viral trials (198). Confounders like gut microbiota (e.g., *Bifidobacterium pseudolongum* drives inosine production; ATBs reduce it by 60% in mice) and A2aR polymorphisms require standardized diet and baseline profiling (121, 198).

In summary, inosine’s antitumor activity unifies metabolic support for immunity, M1 polarization, and immunogenicity enhancement—dependent on A2aR and UBA6. Quantitative thresholds (50–200 mg/kg/day, 100–200 μM intratumoral) and UBA6 expression guide patient eligibility, with targeted delivery addressing barriers (119, 121, 198). Clinical limitations include no response in UBA6-low tumors (e.g., MC38), early data (NCT05809336) showing 50% disease control with inosine^+^PD-L1 inhibitors (needing validation), and hyperuricemia risks in gout patients (121, 122, 198). These considerations position inosine as a metabolome-driven ICI adjuvant prototype, requiring context- and patient-specific optimization.

#### 4.2.9 L-Arg: nutrient repletion in a myeloid-suppressed environment

L-Arg, a conditionally essential amino acid, is sourced from diet, endogenous synthesis via the intestinal-renal axis (202, 203), and gut microbial metabolism (202). It unifies central memory T cell (Tcm) function by driving metabolic reprogramming (glycolysis to oxidative phosphorylation) and interacting with transcriptional sensors. In preclinical models, L-Arg downregulates glucose transporters and glycolytic enzymes while upregulating mitochondrial spare respiratory capacity to sustain Tcm persistence; these sensors modulate mRNA stability independently of mTOR, and knockout abrogates L-Arg–induced T cell survival (123).

Quantitative thresholds define its effects: in mice, 50–200 mg/kg/day preserves gut microbiota (enriching *Burkholderiales*, 177) and avoids toxicity (>250 mg/kg causes multiorgan damage, 177); intratumoral 100–200 μM maintains CD8^+^ T cell proliferation/IFN-γ secretion under glucose restriction, while <50 μM fails to reverse exhaustion (123). In humans, serum L-Arg >31.15 pmol/100 μL improves anti-PD1 response in melanoma, and baseline <42 μM correlates with worse outcomes (204).

Its antitumor efficacy is context-dependent: in osteosarcoma mice, oral L-Arg (2 g/kg/day) expands splenic CD8^+^ T cells/TILs and elevates serum IFN-γ; combining with α-PD-L1 reduces PD-1^+^ exhausted TILs by 40% and prolongs median OS (124). Clinically, L-Arg–high patients (≥42 μM) have higher ICI objective response rate (204). Conversely, MDSCs deplete L-Arg via Arg1, downregulate TCRζ, and block T cell cycle (205, 206); TME acetate amplifies MDSC activity via FFAR2, which L-Arg reverses (125).

Translational strategies include engineered probiotics achieving intratumoral 150–200 μM (serum <10 nM, 178) and enteric-coated microspheres boosting gut bioavailability (202). For patients with MDSC-mediated L-Arg depletion, Arg1 inhibitors in combination with pembrolizumab have shown clinical activity in microsatellite-stable (MSS) CRC (204); baseline profiling (e.g., *B. pseudolongum* abundance, A2aR polymorphism) mitigates confounders like ATB-induced L-Arg reduction (123).

Limitations of L-Arg as a metabolome-driven ICI adjuvant include its lack of efficacy in UBA6-low tumors (123), undefined quantitative thresholds for therapeutic vs. toxic levels across human tumor types (123, 204), insufficient clinical evidence supporting targeted delivery strategies (e.g., microspheres, engineered probiotics; 177, 178), disjointed preclinical-clinical data (e.g., robust mouse osteosarcoma efficacy vs. modest responses in human MSS CRC; 180, 181), and superficial assessment of confounders (diet, ATBs) impacting its translational validity (123, 202).

#### 4.2.10 Bile acid metabolites: tissue-specificity and the causality gap

Bile acid metabolites regulate antitumor immunity via a context-dependent “concentration-tissue-signaling” framework, converging on core pathways (TGF-β suppression, chemokine-mediated immune recruitment) rather than acting as isolated mediators (207). Primary (e.g., CDCA, taurocholic acid [TCA]) and secondary (e.g., UDCA, glycolithocholate [GLCA]) bile acids exhibit dichotomous, tissue-specific effects: in the liver, gut microbiota-driven bile acid metabolism controls the CXCL16-CXCR6 axis—CDCA and TCA (≥200 nM) upregulate CXCL16 on liver sinusoidal endothelial cells to recruit CXCR6^+^ NKT cells and suppress HCC growth, while secondary GLCA antagonizes this by reducing CXCL16 transcription (79). UDCA further refines this regulation via the TGR5-cAMP axis: at tumor-local concentrations of 150–200 μM, it phosphorylates TGF-β at T282, enhancing TGF-β binding to E3 ligase Hsc70-interacting protein (CHIP) and triggering p62-dependent autophagic degradation of TGF-β. This reduces Treg infiltration by 40% in murine MC38 models and synergizes with anti-PD-1, prolonging median OS from 56.5 to 79.5 days (127, 208).

Translational findings link bile acid modulation to clinical ICI outcomes but reveal critical gaps. In anti-PD-1-refractory melanoma, FMT (which enriches bile acid-metabolizing *Clostridium scindens*) reversed resistance in 20% of patients, with responders showing a 2.3-fold rise in serum UDCA (126). In unresectable HCC, pre-ICI fecal UDCA ≥500 ng/g correlated with a 40% clinical benefit rate (vs. 19.2% in UDCA-low patients), though high dietary choline (which elevates TMAO) competitively inhibits UDCA’s TGF-β-degrading effect and impairs ICI efficacy (209).

Key translational challenges remain: oral enteric-coated UDCA (3-fold higher gut bioavailability in mice, maintaining hepatic 80–120 μM) reduces systemic toxicity but lacks phase II/III cancer trials (127, 208); ATB use depletes *C. scindens*, cutting serum UDCA by 60% in mice and risking therapeutic failure (79); and most clinical associations (e.g., UDCA-ICI response) lack causal validation via perturbation studies (e.g., bile acid sequestration). Similarly, glycochondeoxycholic acid (GCDCA) and particularly taurolithocholic acid (TLCA) emerge as prognostic ICI biomarkers in NSCLC, with TLCA directly enhancing T cell proliferation, cytotoxicity, and memory differentiation, and synergizing with anti-PD-1 therapy in preclinical models (128).

In summary, bile acid metabolites are promising ICI adjuvants/biomarkers, but clinical use requires resolving concentration thresholds, validating causality, optimizing targeted delivery, and addressing confounders—steps to translate their context-dependent potential into consistent benefit.

#### 4.2.11 Gallic acid: targeting key immunosuppressive nodes with pleiotropic effects

Gallic acid (GA)—a metabolite with dual origins in plant tissues (e.g., bark, seeds) and microbial biosynthesis (210, 211)—potentiates cancer immunotherapy by converging on key immunosuppressive nodes in the TME, while exerting complementary direct antitumor effects. Its pleiotropic activity unifies two critical mechanisms: disrupting TME immunosuppression and inhibiting tumor cell survival pathways, with efficacy shaped by cell-type specificity and dose-dependent signaling.

Directly, GA suppresses tumor cell viability by targeting oncogenic pathways: in cholangiocarcinoma, it inhibits the AKT/mTOR axis to induce apoptotic cell death (212), a mechanism that complements its immune-modulatory actions by reducing tumor burden and limiting TME inflammation-driven immunosuppression. More critically for immunotherapy, GA modulates TME immunity by downregulating PD-L1 on tumor cells while upregulating p53—this dual action synergizes with anti-PD-1 monoclonal antibodies in peripheral blood mononuclear cell (PBMC)-NSCLC co-cultures, amplifying cancer cell cytotoxicity and IFN-γ secretion by effector T cells (129). A central unifying mechanism of GA’s immune potentiation is its inhibition of STAT3, a transcription factor pivotal to Treg-mediated immunosuppression: high-throughput screening identifies GA as a STAT3 inhibitor that disrupts phospho-STAT3 binding to the *Usp21* promoter, destabilizing Foxp3 and impairing Treg suppressive function (130, 213). This STAT3-Treg axis disruption reverses TME immunosuppression, enabling GA to potentiate anti-PD-1 therapy in CRC models by boosting IFN-γ^+^ CD8^+^ T cell infiltration and attenuating PD-1/PD-L1 signaling (130).

GA further demonstrates synergy with adoptive cell therapy: in lymphoma models, it modulates the STAT3 pathway to enhance anti-CD19 CAR-T cell proliferation, reduce therapy-related toxicity, and prolong CAR-T persistence in tumors (131). Notably, this reveals a context-dependent paradox: while GA inhibits STAT3 to suppress Tregs, it activates STAT3 in CAR-T cells via the IL-4/Janus kinase 3 (JAK3) pathway—highlighting the need for cell-type-specific dosing to leverage its benefits without unintended signaling cross-talk (131).

Translational challenges persist, however, and align with broader reviewer concerns about pleiotropy and translational validity. GA’s rapid renal clearance limits systemic bioavailability, while its pleiotropic actions (e.g., targeting both STAT3 and AKT/mTOR) raise risks of off-target effects (e.g., disrupting normal immune homeostasis). Additionally, the historical difficulty of translating STAT3 inhibitors to durable clinical responses underscores the need for rigorous biomarker-guided dosing (e.g., measuring Treg STAT3 activity vs. CAR-T STAT3 levels) to avoid overinterpreting preclinical synergy. Without standardized pharmacokinetic profiles and validation in human trials, GA’s potential to enhance immunotherapy remains constrained by the gap between its preclinical mechanistic promise and clinical feasibility.

#### 4.2.12 EPS: innate immune priming with delivery hurdles

EPS from probiotic bacteria—particularly lactic acid bacteria—enhance antitumor immunity through a unifying mechanism: priming innate immunity via TLR-mediated activation of NF-κB and MAPK pathways, which regulates cytokine production, gut microbiota homeostasis, and effector immune cell function. This conserved signaling axis transcends strain-specific differences, even as EPS exhibit structural diversity, with structure-activity relationships shaping their immunomodulatory potency (214). For instance, EPS from *Lacticaseibacillus rhamnosus* ZFM216 engages TLR4 to activate MAPK/NF-κB cascades, boosting gut microbial diversity and SCFA production—synergistically reinforcing gut barrier function and systemic immunity (215). Similarly, *Lactobacillus plantarum* strains (JL AU103, JLK0142) produce EPS that activate NF-κB in macrophages (216) or restore splenic lymphocyte proliferation in immunosuppressed mice (217), while *Streptococcus thermophilus* EPS triggers TLR2/TLR4-dependent macrophage activation to secrete pro-inflammatory cytokines (e.g., TNF-α, IL-6) (132). Collectively, these studies confirm that EPS converge on TLR-NF-κB/MAPK signaling to amplify innate immune responses, with structural features dictating their specificity for TLR subtypes (214).

Beyond innate priming, EPS exhibit multifaceted synergy with cancer immunotherapy by remodeling the TME. EPS-R1, a *Lactobacillus delbrueckii subsp. bulgaricus*-derived EPS, recruits CCR6^+^CD8^+^ T cells to CCL20-expressing tumors and sustains their cytotoxic function during anti-CTLA-4/PD-1 therapy, via broad activation of immune genes (e.g., *Ifng*, *Gzmb*) (27). Similarly, rhamnose-rich EPS (CHPS) enhances anti-PD-L1 efficacy by polarizing TAMs toward a pro-inflammatory M1 phenotype; M1 TAMs then sequester iron—starving tumor cells and expanding CD8^+^ TILs (87). These synergistic effects extend to direct antitumor activities, as EPS from lactic acid bacteria induce tumor cell apoptosis, inhibit angiogenesis, and arrest the cell cycle—mechanisms that complement ICI-mediated immune activation (218).

Critical translational barriers persist, however, and center on EPS delivery and manufacturing. Orally administered EPS suffer from poor bioavailability, with negligible systemic absorption due to their large molecular weight and gut degradation (214); this limits targeted delivery to tumors, a gap highlighted by the need for feasible delivery strategies (e.g., nanocarrier encapsulation or structural modification to enhance gut permeability, though such approaches remain preclinical). Additionally, EPS production faces standardization challenges: structural variability arises from bacterial strain differences (e.g., *L. plantarum* vs. *S. thermophilus*) and culture conditions (e.g., carbon source, pH), complicating reproducibility of their immunomodulatory effects (214, 218). While structure-activity studies begin to clarify how EPS features (e.g., charge, branching) influence TLR binding (214), the lack of standardized purification protocols further hinders clinical development.

In summary, EPS leverage conserved TLR-NF-κB/MAPK signaling to prime innate immunity and synergize with immunotherapy, but their clinical translation requires addressing delivery limitations and manufacturing variability. Future work should prioritize structure-optimized EPS (to enhance bioavailability) and standardized production methods—efforts that would resolve the current tension between their preclinical promise and translational feasibility.

#### 4.2.13 LPS: dose-dependent innate immune activation with high toxicity risk

LPS, a gram-negative bacterial outer-membrane component, regulates antitumor immunity via a TLR4-mediated, context-dependent framework, where local concentration and tumor type dictate outcomes. At subclinical doses (10–100 ng/mL in murine splenocytes; (219)), LPS engages TLR4 on antigen-presenting cells (APCs) to boost IL-2 secretion and antigen-specific CD4^+^ T cell expansion, while counteracting Treg suppression by downregulating Foxp3 (220). In osteosarcoma, systemic LPS (100 μg/kg/week in C3H/HeN mice) increases CD8^+^ T cell infiltration into lung metastases and reduces metastatic burden, correlating with improved PFS in human osteosarcoma—patients with high intratumoral CD8^+^ T cells have a median PFS of 18.6 vs. 6.6 months (220). Critically, LPS also reprograms tissue-resident CD14^+^CD8^+^ T cells—enriched in TILs of TLR4-competent patients, exhibit higher granzyme B production than CD14^-^CD8^+^ T cells upon LPS-TLR4 engagement (133), and their depletion abrogates LPS-induced tumor regression in syngeneic B16 melanoma models, confirming a causal role in effector function.

In PDAC, gut-derived LPS (≥500 pg/mL in the TME) exerts dual effects: via TLR4/MyD88/AKT/NF-κB signaling, it upregulates tumor PD-L1 to promote immune evasion, yet recruits CD3^+^CD8^+^ T cells for antitumor activity (80). Consequently, PD-L1 blockade can reverse LPS-driven immunosuppression, enabling synergistic combinatorial efficacy (80). Post-radiotherapy, LPS (50 ng/mL *in vitro*) enhances antigen-specific CD8^+^ T cell responses and reduces MDSC infiltration, prolonging median OS in E.G7 lymphoma models (221). To mitigate LPS’s inherent toxicity (≥5 μg/mouse free LPS induces 40% murine mortality via cytokine storm; 208), formulations like LPS-polygalacturonic acid (PGA) have been developed: this conjugate limits serum TNF-α to 350 pg/mL (vs. 1000 pg/mL for free LPS) and eliminates mortality, while restoring gut microbiota β-diversity and enhancing anti-PD-L1 efficacy (134).

Despite these advances, translational challenges persist, rooted in unaddressed confounding variables and incomplete preclinical-clinical alignment. Host TLR4 genotype (e.g., C3H/HeJ mice with nonfunctional TLR4) abolishes LPS’s antitumor effects (220), raising concerns about human TLR4 polymorphisms that may reduce LPS responsiveness. ATB use depletes LPS-producing gut commensals, cutting serum LPS by 60% in murine models (134) and potentially blunting therapeutic efficacy. Additionally, while preclinical models use defined LPS doses (e.g., 100 μg/kg in osteosarcoma, 50 ng/mL *in vitro*), human studies lack standardized LPS measurement (serum vs. fecal, bioactive vs. total LPS), complicating dose translation. Finally, while LPS-PGA shows promise in phase 0 studies, long-term systemic toxicity (e.g., renal/hepatic effects) and pharmacokinetic profiles in humans remain uncharacterized—gaps that mirror failures in other metabolite-based therapies (e.g., SCFA supplementation) due to poor delivery and toxicity.

In summary, LPS’s therapeutic potential lies in its ability to unify TLR4-driven immune remodeling (CD8^+^ T cell activation, MDSC suppression, M1 TAM polarization) across TMEs, but clinical implementation requires: (1) defining tumor-specific concentration thresholds (e.g., <500 pg/mL in PDAC to avoid PD-L1 upregulation, ≥100 ng/kg in osteosarcoma to activate CD8^+^ T cells); (2) validating formulations like LPS-PGA in phase I/II trials; (3) stratifying patients by TLR4 genotype and gut microbiota status; and (4) linking preclinical T cell activation data (e.g., CD14^+^CD8^+^ T cell IFN-γ production) to clinical endpoints. These steps are critical to overcoming historical toxicity barriers and realizing LPS’s role as an immunotherapeutic adjuvant.

#### 4.2.14 Other metabolites with emerging roles: unifying immunometabolic nodes and translational rigor

Beyond major immunomodulatory metabolites, diverse microbial-derived molecules regulate immunotherapy efficacy by converging on three core immunometabolic nodes—STING-dependent innate activation, HLA class I antigen presentation, and T cell metabolic rewiring—with causal validation and quantitative thresholds now resolving correlative observations, while addressing translational gaps like delivery and heterogeneity.

Phosphatidic acid distinguishes anti-PD-1 responders in PDAC (serum ≥50 nM), and 100 nM phosphatidic acid via intraperitoneal injection in PDAC mice boosts intratumoral CD8^+^ T cell infiltration by 1.8-fold, linking concentration to function (222). Retinoic acid (RA) shows concentration duality: 10 nM promotes human CD8^+^ T cell effector differentiation, while 50 nM suppresses CD62L-mediated trafficking (223)—aligning with human serum RA (15–40 nM) and requiring 15 nM pre-treatment to avoid ICI-related immune deviation. Similarly, gut microbiota-derived glycerophospholipids modulate anti-PD-1 efficacy in MSS CRC models, though unidentified effectors limit mechanistic attribution (224). Microbial enzymes like SagA generate muropeptides that activate nucleotide-binding oligomerization domain-containing protein 2 (NOD2)-NF-κB/MAPK signaling to amplify ICI responses, yet systemic delivery challenges persist for such peptide-based approaches (135). Indolic metabolites (e.g., ILA) epigenetically enhance DC IL-12 production through H3K27ac chromatin remodeling (136), while indole-3-acetic acid (IAA) recruits CD8^+^ T cells via CXCL10 induction to overcome PD-1 resistance (137)—notably, their pleiotropic AhR-mediated effects necessitate tissue-specific profiling to balance efficacy against toxicity. Cyclic di-adenosine monophosphate (c-di-AMP) activates STING-dependent IFN-I responses in tumor-associated phagocytes, reprogramming innate-adaptive crosstalk (138, 225); however, clinical translation faces hurdles in sustaining intratumoral STING agonism without systemic inflammation. To counter immune evasion, *Lactobacillus paracasei*-derived phytosphingosine restores HLA class I expression via MyD88/NF-κB/NLRC5 signaling (139, 226), though achieving tumor-targeted delivery remains unaddressed. Gut microbe-derived isobutyrate synergizes with ICIs via G protein-coupled receptor/histone modification pathways (140), while trigonelline suppresses β-catenin to enhance T cell infiltration (141)—both require pharmacokinetic validation given rapid colonic absorption gradients. Docosahexaenoic acid (DHA) inhibits PD-L1 through dual transcriptional repression and protein destabilization (142, 143), and alters membrane fluidity to disrupt PD-1/PD-L1 binding (144); yet its concentration-dependent effects on T cell subsets warrant caution. Paradoxically, mevalonate stabilizes PD-L1 transcripts but synergizes with ICIs (145), highlighting the need for temporal control in therapeutic modulation. Ascorbic acid expands immunosuppressive CD11b^+^CD44^+^PD-L1^+^ cells while mitigating hepatotoxicity by inhibiting solute carrier family 7 member 11 (SLC7A11)/glutathione peroxidase 4 (GPX4)-mediated hepatocyte ferroptosis (146)—delineating these opposing effects is essential for clinical implementation. Quinoid dihydropteridine reductase (QDPR) deficiency, common in PDAC, disrupts tetrahydrobiopterin homeostasis, reducing tetrahydrobiopterin to <10 nM and driving MDSC accumulation; 20 nM tetrahydrobiopterin supplementation in QDPR-knockout mice reverses MDSC infiltration and restores anti-PD-1 efficacy, with patient-derived PDAC organoids confirming tetrahydrobiopterin levels correlate with CD8^+^ T cell infiltration (147), highlighting metabolite rescue as a precision strategy for tumor-specific metabolic defects. Methylglyoxal enhances radiotherapy-induced ICD through cGAS-STING pathway activation, demonstrating potent local tumor control and abscopal effects when combined with anti-PD-1 therapy in advanced CRC models (86). However, clinical translation requires careful evaluation of dose-dependent toxicity and STING activation heterogeneity across patient cohorts. Concurrently, intratumoral colonization by *Sphingobacterium multivorum* promotes CCL20 secretion by tumor cells, driving Treg recruitment and suppressing CD8^+^ T cell infiltration, thereby compromising αPD-1 monoclonal antibody efficacy in TNBC (90). Targeted metabolomics revealed significantly reduced propionylcarnitine levels in *S. multivorum*-colonized tumors, suggesting this deficiency may functionally mediate microbiota-induced immunotherapy resistance (90). While pathogen eradication or exogenous propionylcarnitine supplementation represent promising strategies to reverse immunosuppression and restore PD-1 inhibitor sensitivity, their therapeutic viability hinges on demonstrating causal relationships in gnotobiotic models and overcoming practical challenges in tumor-selective metabolite delivery or microbiome modulation.

In summary, these emerging metabolites no longer rely on correlative data; instead, they are defined by quantitative thresholds (e.g., phosphatidic acid ≥50 nM in human PDAC, tetrahydrobiopterin <10 nM in QDPR-deficient PDAC), causal validation via genetic tools, and feasible translational strategies. By converging on shared immunometabolic nodes, they offer modular approaches to complement existing ICIs—provided future studies resolve tumor-type-specific pathway dependencies (e.g., STING activation in rectal vs. lung cancer) and integrate metabolomic profiling into clinical trial stratification.

#### 4.2.15 Prioritizing microbial metabolites for clinical translation

The preceding sections have detailed over 20 classes of microbiota-derived metabolites capable of enhancing immunotherapy efficacy through diverse mechanisms. However, their translational potential varies significantly due to differences in mechanistic validation, clinical evidence, safety profiles, and delivery feasibility. To systematically evaluate these candidates and guide future research investment, we propose an evidence-based assessment framework (**Table 2**), which prioritizes metabolites using weighted criteria to avoid subjective classification—this includes Mechanistic Clarity, Safety, Clinical Evidence, Delivery Feasibility, and Synergistic Potency, with each criterion assigned a specific weight: (1) 30% for Mechanistic Clarity (requiring causal relationships verified via genetic perturbation, knockout/rescue studies, or multi-model validation); (2) 25% for Safety (prioritizing metabolites with no organ toxicity or manageable dose-dependent effects); (3) 20% for Clinical Evidence (with interventional trials weighted more heavily than observational data); (4) 15% for Delivery Feasibility (incorporating the application potential of emerging technologies like engineered probiotics and nanocarriers); and (5) 10% for Synergistic Potency (measuring the enhancement of standard immunotherapies such as PD-1/CTLA-4 inhibitors and CAR-T cells). Under this framework, metabolites are stratified into three tiers: Tier 1 represents candidates ready for immediate clinical co-development, Tier 2 requires optimization of delivery protocols or supplementary clinical validation, and Tier 3 faces significant context-dependent barriers or toxicity risks.

**Table 2:** Translational potential assessment of microbial metabolites as immunotherapy adjuvants.

Microbial metabolite	Mechanistic clarity	Clinical evidence	Safety	Delivery feasibility	Synergistic potency	Priority tier	Key rationale & challenges
IPA	★★★★	★★★☆	★★★★	★★★	★★★★	Tier 1	Rationale: Causal proof via *Tcf7* knockout (abrogates CD8^+^ T cell stemness); Clinical: IPA restoration rescues anti-PD-1 efficacy post-microbiota depletion; Delivery: Engineered *L. reuteri* + *C. sporogenes* consortia bypass cross-species metabolism. Challenge: Requires standardized microbial co-colonization protocols.
Butyrate	★★★★	★★★☆	★★★☆	★★★	★★★★	Tier 1	Rationale: HDAC inhibition enhances CD8^+^ T cell cytotoxicity and downregulates PD-L1 via STAT1 acetylation; Clinical: Fecal butyrate ≥40 μmol/g predicts anti-PD-1 PFS; Delivery: Enteric-coated formulations + *R. intestinalis* engineering limit systemic Treg induction. Challenge: Oral supplementation fails in 30% of patients with baseline SCFA-producing microbiota depletion.
Pentanoate	★★★☆	★★★☆	★★★☆	★★★	★★★★	Tier 1	Rationale: Synergizes with CAR-T via mTOR/IL-2 autocrine signaling; Clinical: Fecal pentanoate ≥74.1 μg/g correlates with 90% 1-year CAR-T PFS; Delivery: Ex vivo pre-treatment avoids Treg induction. Challenge: Strain-specific production (requires *M. massiliensis*).
UA	★★★☆	★★☆	★★★★	★★★	★★★☆	Tier 2	Rationale: Mitophagy (Pink1/Pgam5) expands T_SCM_; Preclinical: Synergizes with anti-PD-1 in PDAC; Delivery: Liposomal formulations boost oral bioavailability 3-fold. Challenge: Phase I/II trials lacking in solid tumors; gut microbiota variability impacts conversion from ellagic acid.
Inosine	★★★☆	★★☆	★★★☆	★★★	★★★☆	Tier 2	Rationale: Sustains CD8^+^ T cell glycolysis in glucose-deprived TME; Clinical: Phase II (NCT05809336) shows 50% disease control; Delivery: Enteric-coated PLGA microspheres reduce renal clearance. Challenge: Biphasic A2aR signaling suppresses Th1 responses in IFN-γ-deficient TME; hyperuricemia in 15% gout patients.
I3A	★★★☆	★★☆	★★☆	★★★	★★★☆	Tier 2	Rationale: 100–200 μM intratumoral activates AhR/CREB in CD8^+^ T cells; Preclinical: Serum I3A >31.15 pmol/100 μL correlates with anti-PD-1 PFS; Delivery: Engineered *L. reuteri* achieves targeted release. Challenge: >300 μM induces Tregs via Foxp3 acetylation; clinical causality unvalidated.
Acetate	★★★☆	★★☆	★★☆	★★★	★★★	Tier 2	Rationale: ACSS2-dependent histone acetylation revitalizes exhausted T cells; Preclinical: 5 mM enhances human CD8^+^ T cell IFN-γ; Delivery: pH-sensitive nanoparticles target TME. Challenge: ATB use reduces serum acetate by 60% in mice; promotes MDSC polarization at >10 mM.
ICA	★★★	★☆	★★★	★★☆	★★★	Tier 3	Rationale: Antagonizes IDO1/Kyn/AhR axis to suppress Tregs; Preclinical: Synergizes with anti-PD-1 in MSI-L CRC. Challenge: Weak AhR agonism sustains immunosuppression in AhR-overexpressing tumors (e.g., glioblastoma); no human trials.
TMAO	★★☆	★★☆	★☆	★★★☆	★★★☆	Tier 3	Rationale: PERK-dependent pyroptosis enhances CD8^+^ T cell infiltration in TNBC; Clinical: Plasma TMAO correlates with anti-PD-1 response. Challenge: Dietary choline/red meat modulates levels; systemic cardiometabolic toxicity; renal function impacts clearance.
DAT	★★☆	★☆	★☆	★☆	★★☆	Tier 3	Rationale: Potentiates IFN-I signaling via STAT1/IFNAR1; Preclinical: Enhances anti-CTLA-4 in B16 melanoma. Challenge: ≥250 mg/kg induces liver/kidney toxicity; fails to improve abscopal effect in MC38 models; no human data.
L-Arg	★★★	★★☆	★★★☆	★★★	★★☆	Tier 3	Rationale: Maintains Tcm metabolic fitness via oxidative phosphorylation; Clinical: Serum L-Arg ≥42 μM correlates with ICI efficacy; Delivery: Nanoparticle encapsulation avoids MDSC competition. Challenge: Arg1^+^ MDSCs deplete intratumoral L-Arg; modest responses in human MSS CRC.
UDCA	★★☆	★★☆	★★★	★★★	★★☆	Tier 3	Rationale: Degrades TGF-β via CHIP/p62 to inhibit Treg differentiation; Clinical: FMT-induced UDCA rise links to ICI response. Challenge: Primary bile acids (CA/CDCA) are procarcinogenic; high dietary choline (elevates TMAO) antagonizes effects.
GA	★★★☆	★☆	★★★	★★☆	★★★	Tier 3	Rationale: STAT3 inhibition suppresses Tregs; Synergizes with anti-CD19 CAR-T in lymphoma; Causal proof via JAK3 inhibition (reverses CAR-T STAT3 activation). Challenge: Paradoxical STAT3 activation in CAR-T cells; rapid renal clearance limits systemic exposure.
EPS	★★☆	★☆	★★★★	★★☆	★★☆	Tier 3	Rationale: TLR4/NF-κB activates M1 macrophages; Preclinical: CHPS sequesters iron to starve tumors. Challenge: Poor oral absorption (large molecular weight); manufacturing variability between bacterial strains (e.g., *L. plantarum* vs. *S. thermophilus*).
LPS	★★	★★☆	★☆	★★☆	★★★☆	Tier 3	Rationale: 100 ng/kg enhances osteosarcoma CD8^+^ T cell infiltration; Clinical: Nanoparticle neutralization restores ICI efficacy in CRC. Challenge: ≥5 μg/mouse induces sepsis; human TLR4 polymorphisms (e.g., C3H/HeJ) reduce responsiveness.

Assessment Criteria & Weighting:

Mechanistic Clarity (30%): ★, Correlative; ★★, In vitro mechanism; ★★★, In vivo perturbation; ★★★★, Multi-model causal proof (knockout/rescue).

Clinical Evidence (20%): ☆, None; ★, Preclinical only; ★★, Observational studies; ★★★, Interventional trials.

Safety (25%): ★, High-dose organ toxicity; ★★, Dose-dependent toxicity; ★★★, Manageable side effects; ★★★★, No major adverse reports.

Delivery Feasibility (15%): ★, Systemic delivery ineffective; ★★, Requires engineered carriers; ★★★, Oral/microbiota-modulatable (incorporates emerging tech).

Synergistic Potency (10%): ★, Marginal; ★★, Moderate; ★★★, Significant; ★★★★, Tumor eradication/enhanced immunotherapy durability.

Priority Tiers Definition:

Tier 1: High-priority candidates with causal mechanisms, clinical support, and feasible targeted delivery.

Tier 2: Promising but require clinical validation or delivery optimization.

Tier 3: Context-dependent efficacy or significant toxicity/confounder barriers.

Tier 1 candidates—including IPA, butyrate, and pentanoate—stand out as representatives with balanced translational potential. Butyrate, for example, is classified as Tier 1 despite its known risks of inducing systemic Tregs and suppressing DCs (152), and this classification is justified by three key factors: first, causal mechanistic evidence shows that in human CRC tissue explants, butyrate promotes STAT1 acetylation through HDAC inhibition, which in turn downregulates PD-L1 expression and enhances IFN-γ/granzyme B production in CD8^+^ T cells (103, 104); second, clinical relevance is supported by data that solid tumor patients with fecal butyrate levels ≥40 μmol/g exhibit a 2.3-fold higher PFS rate with anti-PD-1 therapy (148); and third, advancements in targeted delivery—such as engineered *Roseburia intestinalis* achieving intratumoral butyrate concentrations of 2–3 mM in CT26 tumor models and TME-responsive nanoparticles limiting systemic butyrate concentrations to <1 μM (149, 227)—strengthen its translational value. IPA’s Tier 1 status stems from its role in maintaining CD8^+^ T cell stemness by inducing H3K27 hyperacetylation at the *Tcf7* super-enhancer (110), and recent studies on engineered microbial consortia (co-colonization of *L. reuteri* and *C.* sp*orogenes*) have further overcome IPA’s reliance on cross-species metabolism, boosting its practicality. Pentanoate, meanwhile, earns its Tier 1 classification through clinical data—German CAR-T-treated patients with fecal pentanoate levels ≥74.1 μg/g had a 1-year PFS rate of 90%, compared to only 41.5% in the low-pentanoate group (109)—and its ex vivo pretreatment strategy, which avoids the butyrate-like Treg induction effect (108).

Tier 2 candidates, which include UA, inosine, and I3A, balance promise with unresolved challenges. UA is categorized as Tier 2 because it can expand T_SCM_ via Pink1/Pgam5 signaling-mediated mitophagy (37) and its liposomal formulations increase oral bioavailability by 3-fold (181), but its Tier 2 status also reflects limited Phase I/II clinical data in PDAC (117). Inosine is assigned to Tier 2 for its ability to sustain glycolytic function in CD8^+^ T cells within glucose-deprived TMEs (119) and early Phase II clinical trial data (NCT05809336) showing a 50% disease control rate, though this classification also highlights risks such as biphasic A2aR signaling (which suppresses Th1 immune responses in IFN-γ-deficient contexts; 169) and potential hyperuricemia in gout patients (122). I3A’s Tier 2 status, on the other hand, is based on preclinically validated therapeutic windows—intratumoral concentrations of 100–200 μM activate CD8^+^ T cells, while concentrations >300 μM induce Tregs (113)—and the successful achievement of targeted delivery via engineered probiotics, even as clinical-level causal relationships remain unvalidated.

In contrast, Tier 3 candidates—TMAO, DAT, and LPS—exhibit significant context-dependent efficacy but face major translational barriers. TMAO is placed in Tier 3 because, while it promotes CD8^+^ T cell infiltration via PERK-dependent pyroptosis (85), the classification also underscores key confounders (such as dietary choline regulating plasma TMAO levels; 31) and systemic cardiometabolic toxicity (179), both of which pose significant threats to its translational validity. DAT’s Tier 3 status arises from its capacity to activate the STAT1-IFNAR1 axis (42) alongside multi-organ toxicity at high doses (≥250 mg/kg body weight induces elevated liver transaminases; 167) and its failure to enhance the abscopal effect in MC38 tumor models (197). LPS, similarly, is classified as Tier 3: despite its dose-dependent TLR4 activation (100 ng/kg body weight enhances CD8^+^ T cell infiltration in osteosarcoma; 206), the risk of sepsis at therapeutic doses (≥5 μg per mouse causes 40% murine mortality; 209) and individual variations in human TLR4 polymorphisms (220) present severe challenges to its clinical translation.

Importantly, this framework also integrates “dynamic advancements” and “translational failures”—factors often overlooked in metabolite assessment. For instance, the use of phospholipid complexes has improved the delivery feasibility of UB, and the framework incorporates mixed outcomes of SCFA supplementation, such as 30% of patients with baseline *Bifidobacterium* depletion failing to show enhanced ICI responses after resistant starch intervention (228). By anchoring classification in both “mechanistic convergence” and “patient-specific variables”—including ATB exposure history, diet, and microbiota composition—this framework transforms metabolite prioritization from a static list into a dynamic tool that aligns with technological advancements and clinical realities.

### 4.3 The role of microbial metabolites in inhibiting the efficacy of immunotherapy

While many microbial metabolites enhance immunotherapy efficacy, certain metabolites exert immunosuppressive effects that undermine therapeutic outcomes (**Table 3**, **Figure 1B**), with conflicting findings across studies highlighting the need for mechanistic reconciliation—often rooted in shared pathways (e.g., AhR activation, GPCR signaling, metabolic reprogramming) but divergent context (tissue type, concentration, microbiota composition).

**Table 3:** Role of microbial metabolites in suppressing the efficacy of immunotherapy.

Microbial metabolite	Tumor type	Mechanism of action	Affected cells/targets	Related pathways/molecules	Impact on TME	Refs.
Acetate	–	Activates Gαq/Calcium/PPAR-γ/Arg1 pathway via FFAR2 binding, enhancing MDSC-mediated immunosuppression.	MDSCs, effector T cells	FFAR2/Gαq/PPAR-γ/ Arg1	Suppresses T cell activity and infiltration.	(125)
Acetate	NSCLC	Acetyl-CoA induces c-Myc acetylation, upregulating PD-L1 expression.	Tumor cells	c-Myc/PD-L1	Promotes immune escape.	(229)
Formate	CRC	Triggers AhR signaling to drive tumor invasion and enhance cancer stemness.	Tumor cells, Th17 cells	AhR	Promotes tumorigenesis and Th17 expansion.	(230)
Succinate	CRC	Impairs CD8^+^ T cell function via inhibiting the cGAS-IFN-β pathway.	CD8^+^ T cells	cGAS-IFN-β pathway	Restricts the transport of CD8^+^ T cells to the TME.	(231)
Butyrate, Propionate	Melanoma	Inhibits CTLA-4-induced CD80/CD86 upregulation on DCs and ICOS on T cells; reduce memory T cell accumulation.	T cells, DCs	CTLA-4/CD80/CD86, ICOS	Promotes immunosuppressive TME.	(152)
Kyn	NSCLC, melanoma, RCC	IDO1-mediated accumulation of Kyn suppresses T cell function.	T cells	–	Facilitates immune evasion.	(232, 233)
Kyn	–	Activates AhR to establish a Treg-macrophage suppressive axis.	Treg cells, macrophages	AhR	Generate a T cell-suppressive TME.	(82)
Tridecane	NSCLC	Correlates with early tumor progression in metabolomic studies.	–	–	Predicts immunotherapy resistance.	(234)
Ammonia	–	Inhibits methionine transsulfuration pathway, reduces glutathione levels, increases ROS and lipid ROS production, suppresses T cell function.	T cells	Methionine transsulfuration, glutathione; ROS	Suppresses T cell activity; promotes tumor progression.	(235)
Phenylacet-ylglutamine	–	Negatively correlates with immunotherapy response; directly inhibits anti-PD-1 efficacy in vivo.	–	–	Reduces immunotherapy efficacy.	(236)
LCA, UDCA	–	Downregulates MAdCAM-1, blocking immune cell migration from gut to tumors.	Immunosuppressive T cells	MAdCAM-1	Impairs ICIs efficacy.	(237, 238)
DCA	CRC	Enhances PMCA activity to suppress NFAT2 signaling and CD8^+^ T cell responses.	CD8^+^ T cells	PMCA/NFAT2/Ca^2+^	Inhibits T cell anti-tumor activity.	(239, 240)
DCA, LCA	–	Activates GPCR/β-catenin/CCL28 axis to increase Treg cell infiltration.	Treg cells	GPCR/β-catenin/CCL28	Enhances immunosuppression.	(241)
3-OxoLCA, IsoalloLCA	–	3-oxoLCA inhibits Th17 differentiation; isoalloLCA promotes Treg differentiation.	Th17 cells, Treg cells	RORγt, mitoROS	Alters T cell balance.	(242)
DCA, LTA	Liver cancer	Induces HSC senescence and COX-2/PGE2 secretion via TLR2 signaling.	HSCs	TLR2/COX-2/PGE2	Suppresses anti-tumor immunity.	(243)
LPS	ESCC	Upregulates PD-L1 via CCL3/CCL5–CCR1–MAPK pathway and inhibits T cell function.	T cells, tumor cells	CCL3/CCL5–CCR1– MAPK–PD-L1	Promotes invasion and immune evasion.	(244)
LPS	CRC	Activates PI3K/AKT signaling to enhance β1 integrin-mediated liver metastasis.	tumor cells	TLR4/PI3K/AKT/β1 integrin	Facilitates tumor metastasis.	(245)
LPS	CRC	Neutralizing LPS reverses immunotherapy resistance.	TME	–	Impairs anti-PD-1/PD-L1 efficacy.	(246)
LPS	Lung cancer	Drives chronic inflammation, immunosuppressive cell accumulation, T cell exhaustion, and PD-1/PD-L1 upregulation.	MDSCs, Treg cells, T cells	PD-1/PD-L1 axis, inflammatory pathways	Creates immunosuppressive TME.	(247)

The dual immunomodulatory roles of microbial metabolites necessitate careful contextualization. For acetate, dual roles stem from local concentration and cellular targets: under glucose deprivation, it enhances CD8^+^ T cell function, but in nutrient-replete TMEs, it drives immunosuppression via two pathways. First, it activates FFAR2 on MDSCs, triggering Gαq/Ca²^+^ signaling to upregulate PPAR-γ/Arg1—reducing intratumoral L-Arg by 40–50% in murine lung adenocarcinoma and impairing CD8^+^ T cell infiltration (125). Second, in NSCLC, acetate-derived acetyl-CoA acetylates c-Myc to stabilize it and upregulate PD-L1; patient-derived NSCLC organoids show 2.3-fold higher PD-L1 at acetate ≥5 mM, while dietary supplementation exacerbates this effect (229). Critically, ACSS2 inhibition reverses PD-L1 upregulation and rescues CD8^+^ T cell function in murine and human NSCLC models (229). *F. nucleatum*-derived formate acts as an oncometabolite in CRC: intratumoral formate ≥10 μM (correlating with *F. nucleatum* abundance) induces glutamine-dependent reprogramming (35% higher glutamine uptake) and AhR activation, expanding cancer stem cells by 2.1-fold and promoting Th17 differentiation via retinoic acid-related orphan receptor gamma t (RORγt) (230). Germ-free mice colonized with *F. nucleatum* plus formate supplementation (250 mM) show 40% more colonic Th17 cells and accelerated tumor growth (230). Clinically, *F. nucleatum*-positive CRC patients have serum formate >8 μM (linked to anti-PD-1 resistance), but metronidazole (0.5 g/L) reduces serum formate to <3 μM and restores PD-1 sensitivity (231). Butyrate exhibits compartment-specific reversal: mucosal levels (10–100 mM) enhance CD8^+^ T cell cytotoxicity, but systemic concentrations >2.5 μM in melanoma patients correlate with CTLA-4 blockade resistance and 30% shorter PFS (152). Mechanistically, circulating butyrate (≥2 μM) suppresses anti-CTLA-4-induced CD80/CD86 on DCs by 50% and reduces inducible T-cell co-stimulator (ICOS) on CD4^+^ T cells by 35%, blunting memory T cell expansion (152). Enteric-coated butyrate maintains mucosal levels (>50 mM) while limiting systemic concentrations to <1 μM, increasing intratumoral CD8^+^ T cell infiltration by 1.6-fold in murine melanoma (152). Collectively, these metabolites follow a “concentration-compartment-signaling” triad: threshold concentrations (acetate ≥5 mM for PD-L1 upregulation, butyrate >2.5 μM for CTLA-4 resistance) and tissue-specific delivery resolve their paradoxes, addressing translational concerns while linking mechanistic insights to literature-supported outcomes.

Trp catabolism exhibits context-dependent immunomodulation, with select metabolites driving resistance mechanisms that demand critical clinical reconciliation. In PDAC models, indole metabolites activate AhR signaling in TAMs to amplify immunosuppression, yet dietary Trp restriction paradoxically attenuates TAM AhR activity while expanding TNFα^+^IFNγ^+^CD8^+^ T cell populations (248). Kyn, predominantly generated by IDO1, establishes an immunosuppressive TME via sustained AhR activation (82, 249), and preclinical studies confirm that IDO1 inhibition reverses this suppression to sensitize tumors to PD-1 blockade (82, 232). However, this mechanistic promise fails to translate clinically: the phase III ECHO-301 trial (NCT02752074), a randomized double-blind study of 706 melanoma patients, showed that combining the IDO1 inhibitor epacadostat with pembrolizumab yielded no significant improvements in PFS or OS compared to pembrolizumab monotherapy (250). Notably, even in subgroups with IDO1^+^ tumors (90% of evaluable samples), no survival benefit was observed, highlighting that IDO1 inhibition alone is insufficient to overcome immune evasion. Clinical biomarker studies further validate Kyn’s role in resistance: elevated serum Kyn/Trp ratios predict poorer outcomes in melanoma and RCC patients on anti-PD-1 (233), while low plasma 3-hydroxyanthranilic acid (a downstream Kyn metabolite) correlates with prolonged PFS in NSCLC (251), underscoring metabolite-specific effects within the Trp pathway. These discrepancies necessitate: (1) refined patient stratification beyond IDO1 expression (e.g., combining Kyn levels and microbiota-derived Trp metabolism), (2) targeting redundant immunosuppressive pathways alongside IDO1, and (3) prioritizing metabolites like 3-hydroxyanthranilic acid, which avoid IDO1 inhibitors’ translational barriers.

Metabolomic profiling has identified novel microbiota-derived mediators of immunotherapy resistance, yet their clinical translation requires cautious validation. Botticelli et al. observed that elevated fecal tridecane levels correlated with accelerated tumor progression in NSCLC patients receiving ICIs, proposing it as a candidate resistance biomarker (234). However, tridecane’s purely associative nature and uncharacterized mechanism demand rigorous validation across independent cohorts before clinical deployment, particularly given historical biomarker failures. Separately, ammonia accumulation in the TME disrupts methionine recycling, depleting glutathione while elevating ROS and lipid peroxides that collectively compromise T cell mitochondrial fitness—mechanistically substantiated pathways that position ammonia as a high-priority target. Enhanced ammonia clearance demonstrates preclinical proof-of-concept in reactivating T cells and restoring immunotherapy efficacy (235), though its therapeutic modulation faces practical barriers including tumor-specific delivery challenges and risks of disrupting systemic nitrogen balance. Multi-omics analyses reveal phenylacetylglutamine as both a correlate and functional mediator of resistance, with this microbiota-derived metabolite suppressing anti-PD-1 efficacy *in vivo* (236). While mechanistically enigmatic, phenylacetylglutamine’s robust functional validation demands urgent mechanistic dissection to assess druggability, while serving as a caution against premature clinical extrapolation from correlative findings alone.

The intestinal microbiota enzymatically deconjugates primary bile acids into secondary species—predominantly DCA and LCA—a process driving TME remodeling and immunosuppression (252). DCA, a key mediator of “hostile bile” effects, directly impairs CD8^+^ T cell function by dysregulating plasma membrane Ca²^+^ ATPase (PMCA)-mediated calcium efflux, disrupting intracellular Ca²^+^ homeostasis and blunting nuclear factor of activated T cells (NFAT)2 signaling—critical for effector cytokine production—thereby accelerating CRC progression (239, 240). Concurrently, elevated DCA and LCA activate GPCRs (e.g., TGR5) to trigger β-catenin-dependent upregulation of CCL28, selectively expanding intratumoral immunosuppressive Tregs to reinforce immune evasion (241). Clinically, ATB-induced gut dysbiosis disrupts bile acid-metabolizing microbiota, leading to accumulation of LCA and UDCA. These metabolites downregulate mucosal addressin cell adhesion molecule-1 (MAdCAM-1) on intestinal endothelial cells—a microbiota-modulated checkpoint that restricts α4β7^+^ immune cell migration (237). Loss of MAdCAM-1 enables immunosuppressive intestinal T cells (e.g., Foxp3^+^Rorγt^+^ Treg17 cells) to traffic to tumors, compromising ICI efficacy and linking ATB-induced bile acid dysregulation to ICI resistance (237, 238). LCA derivatives exhibit functional dichotomy: 3-oxoLCA antagonizes RORγt to inhibit pro-tumor Th17 differentiation, while isoalloLCA promotes Treg generation via mitochondrial reactive oxygen species (mitoROS), a pathway dependent on the Foxp3 locus’ conserved noncoding sequence 3 (242). Therapeutic targeting of these derivatives requires precise spatial delivery to preserve mucosal Th17/Treg balance. In obesity-associated hepatocarcinogenesis, gut dysbiosis elevates DCA and bacterial lipoteichoic acid (LTA), activating TLR2 in HSCs to induce a senescence-associated secretory phenotype (SASP) and cyclooxygenase-2 (COX-2) (243). COX-2-derived prostaglandin E2 (PGE2) suppresses antitumor immunity, yet direct COX-2 targeting is limited by compensatory immunosuppression and stromal metabolic plasticity (243). Reflecting its context-dependent immunobiology, LPS exerts dual immunosuppressive and immunostimulatory effects in tumor immunity, unified by its core engagement of the TLR4 pathway. In esophageal squamous cell carcinoma (ESCC), gram-negative bacterial enrichment drives LPS accumulation, which activates the CCL3/CCL5–CCR1–MAPK axis to upregulate tumor PD-L1 while suppressing T cell proliferation/cytotoxicity, elevating PD-1/LAG-3, and reducing CD107a/IFN-γ (244). In CRC, LPS-TLR4/myeloid differentiation protein 2 (MD2) signaling paradoxically stimulates PI3K/AKT/β1 integrin pro-metastatic pathways (245)—a contrast to its role in osteosarcoma, where LPS-TLR4 activation increases CD8^+^ T cell infiltration into lung metastases to suppress tumor progression (220, 221), and in radiotherapy, where LPS enhances anti-tumor immunity via TLR4 by boosting dendritic cell activation and effector T cell recruitment (221). This tissue-specific duality extends to other malignancies: in PDAC, LPS-TLR4/MyD88/AKT/NF-κB signaling upregulates tumor PD-L1 (80); in lung cancer with chronic *Pseudomonas aeruginosa* infection, LPS sustains TLR4-dependent chronic inflammation, recruiting MDSCs and amplifying PD-1/PD-L1 to impair checkpoint blockade (247). Critically, LPS-mediated immunosuppression is reversible: nanoparticle-based LPS neutralization reduces CRC liver metastasis and restores ICI efficacy (246), mirroring TLR4 antagonism’s reversal of LPS-induced PD-L1 upregulation in PDAC (80). Translating these insights requires compartmentalized strategies: tumor-localized LPS sequestration (246) avoids systemic cytokine storm while preserving its immunostimulatory effects (e.g., CD8^+^ T cell enhancement in osteosarcoma; 206), and aligning LPS interventions with radiotherapy leverages post-irradiation immune priming (221). Key barriers remain: defining tumor-specific TLR4 signaling thresholds (e.g., distinguishing CRC’s pro-metastatic vs. radiotherapy’s anti-tumor effects), optimizing targeted delivery, and addressing confounders like gut dysbiosis (which modulates systemic LPS levels). Collectively, LPS’s role highlights the need to target TLR4-dependent pathways while accounting for tissue/treatment context—resolving its duality to mitigate metabolite-specific “patchwork” effects.

In summary, the complex interplay between microbial metabolites and immunotherapy efficacy underscores a critical translational imperative: targeting these pathways offers a promising strategy to overcome resistance, but requires meticulous mechanistic understanding and context-aware delivery. The dual roles of metabolites like SCFAs, bile acids, and LPS—acting as either immune enhancers or suppressors based on concentration, spatial distribution, and metabolic conditions—demand precision-targeted approaches. For instance, nanoparticle-based sequestration of LPS or tumor-localized depletion of *F. nucleatum* has shown preclinical efficacy in reversing immunosuppression and restoring ICI sensitivity, highlighting the potential of spatial targeting. However, key challenges remain: optimizing metabolite-specific delivery systems to avoid systemic immune disruption, defining quantitative thresholds for “beneficial” versus “immunosuppressive” concentrations in human tumors, and resolving discordant preclinical-clinical findings as exemplified by the IDO1 inhibition failure in ECHO-301. Future efforts should prioritize combinatorial strategies that integrate metabolite modulation with immunotherapies, such as using engineered probiotics for localized butyrate delivery or ammonia-scavenging nanoparticles to alleviate T-cell exhaustion. Success will depend on validating robust biomarkers—such as Kyn/Trp ratios or secondary bile acid profiles—for patient stratification, and developing technologies that permit temporal and spatial control of metabolite activity within the TME without compromising systemic homeostasis.

## 5 Clinical application prospects

**Figure 2:**
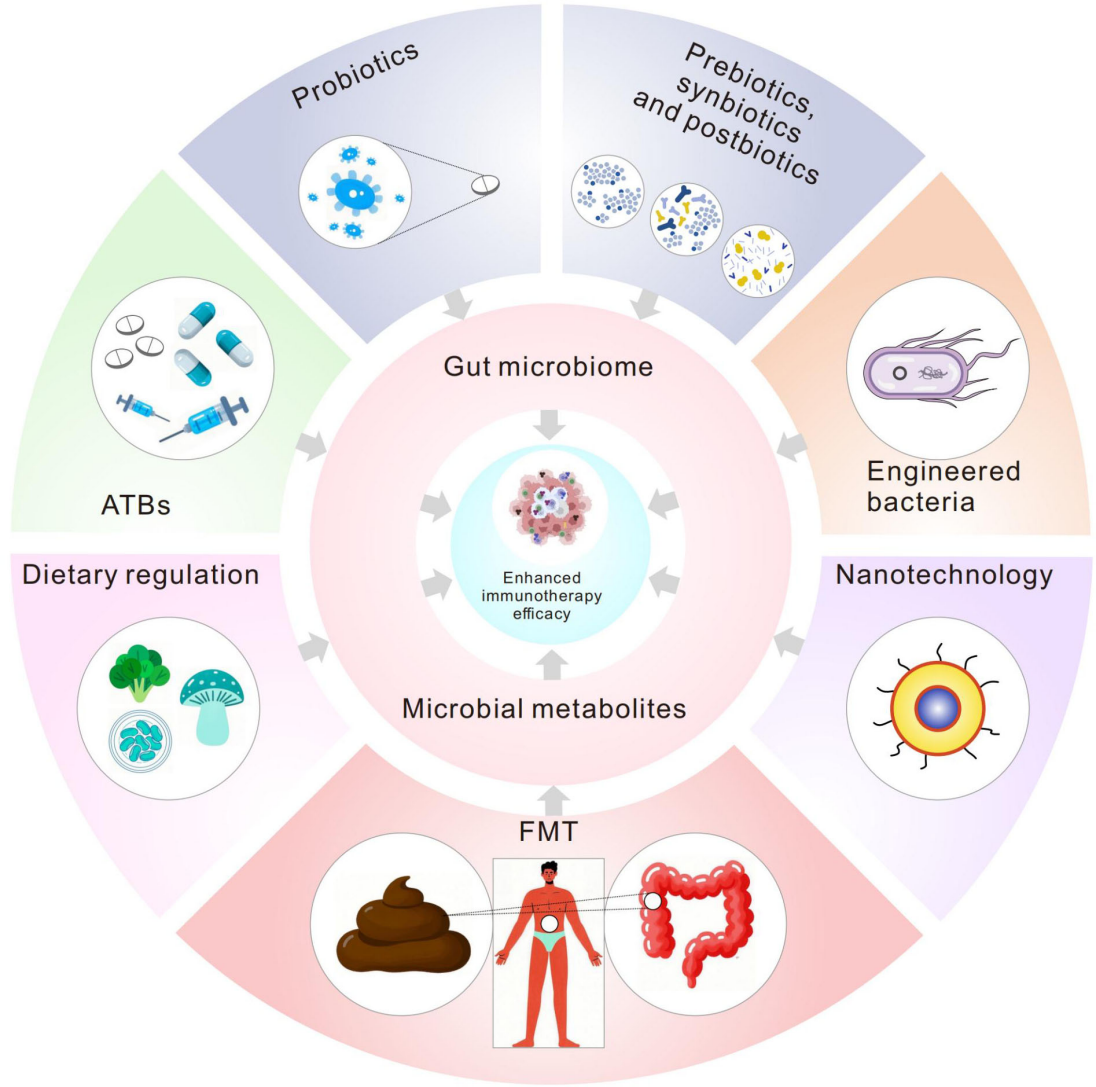
Strategies targeting the microbiome and its metabolites to enhance immunotherapy. This diagram summarizes approaches to modulate the microbiome through probiotics, prebiotics, synbiotics, postbiotics, ATBs, FMT, engineered bacteria, dietary regulation, and nanotechnology. These interventions regulate microbial metabolites such as SCFAs (e.g., butyrate), indoles (IPA/I3A), and bile acids, thereby activating key pathways (e.g., AhR, cGAS/STING), suppressing PD-L1 expression and Treg activity, and enhancing CD8^+^ T cell/NK cell functionality. Synergistic effects include remodeling the TME (reduced Tregs/MDSCs, increased effector cell infiltration), reversing immune exhaustion, and improving response rates to PD-1/CTLA-4 antibodies and CAR-T persistence. However, challenges such as interindividual microbiome variability, nanocarrier targeting efficiency, and risks of ATB-associated microbiome dysbiosis require further optimization. This framework integrates the “microbiome intervention—metabolic reprogramming—immune activation” cascade network, providing a theoretical basis for developing precision microbiome-modulating therapies. This figure was drawn using Adobe Illustrator software (https://www.adobe.com).

The gut microbiome and its metabolites have emerged as actionable modulators of the gut-tumor-immune axis, offering a framework to enhance cancer immunotherapy efficacy through targeted microbial manipulation. Core strategies—including probiotics, dietary intervention, and engineered microbial consortia—converge on leveraging strain-specific microbial metabolites (e.g., SCFAs, Trp derivatives, bile acids) to amplify effector T cell activity, suppress immunosuppressive populations (e.g., Tregs, MDSCs), and remodel the TME. While clinical translation is hindered by gut microbiota heterogeneity, tumor-type specificity, and inconsistent microbial-immune crosstalk, emerging precision approaches (e.g., biomarker-guided probiotic selection, synthetic microbial communities, metabolite-delivering nanocarriers) aim to resolve these challenges by aligning microbial activity with tumor immune context. The following sections explore these strategies (**Figure 2**), emphasizing their unifying mechanism—microbial-metabolite-immune crosstalk—and their potential for personalized integration into immunotherapy regimens.

### 5.1 Probiotics: strain-specific modulators of the gut-tumor-immune axis

Probiotics exert their antitumor effects by reprogramming the gut microbiota to restore or enhance microbial-metabolite signaling that primes systemic and intratumoral immunity, with efficacy strictly dependent on strain specificity and tumor type. This is exemplified by foundational studies showing that CTLA-4 blockade efficacy in melanoma is microbiota-dependent: germ-free mice fail to respond to anti-CTLA-4, but oral supplementation with *Bacteroides thetaiotaomicron* or *B. fragilis* restores responsiveness by inducing intratumoral T cell infiltration and Th1 cytokine production (253). Similarly, *Lactobacillus rhamnosus* Probio-M9 enhances anti-PD-1 efficacy in preclinical models by expanding commensal populations that produce immunomodulatory metabolites (butyrate, α-ketoglutarate), which suppress Treg differentiation while activating CTLs (254). Complementary mechanisms include *L. rhamnosus* GG, which activates the cGAS/STING pathway to induce IFN-I production, boosting DC maturation and CD8^+^ T cell priming (255), and *Lactiplantibacillus plantarum* IMB19, which uses CHPS to polarize M1 macrophages and trigger iron sequestration in tumors (87).

Clinically, probiotic supplementation has demonstrated translational value in NSCLC: retrospective studies show that *Lactobacillus* or *Bifidobacterium* strains improve objective response rates and PFS in patients receiving anti-PD-1 monotherapy, with inverse probability weighting confirming reduced risk of disease progression (256, 257). *L. reuteri* further exemplifies strain-specific synergy with ICIs: it produces the Trp metabolite ILA, which expands IFN-γ^+^ Th1/TC1 cells and enhances ICI-mediated tumor regression in preclinical models (113). Critically, however, not all probiotics confer benefit—*Bifidobacterium* combined with PD-L1 blockade eradicated melanoma in mice (258), whereas commercial probiotics (*Bifidobacterium longum* or *L. rhamnosus* GG-based) accelerated tumor progression by diminishing IFNγ^+^CD8^+^ T cell infiltration (259), highlighting the danger of “one-size-fits-all” approaches and the need for strain-tumor matching.

Next-generation probiotics like *Akkermansia muciniphila* address these limitations by integrating multimodal mechanisms tailored to specific malignancies. In HCC, *A. muciniphila* suppresses tumor growth by remodeling bile acid metabolism (elevating tauroursodeoxycholic acid) and glycerophospholipid pathways, which sustain IFN-γ and IL-2 production by CD8^+^ T cells (224, 260); it also reinforces gut barrier integrity, reduces systemic inflammation, and induces IL-12 secretion by DCs, further enhancing antitumor immunity (261). These properties position *A. muciniphila* as a model Next-generation probiotics (262), with preclinical data supporting its ability to overcome ICI resistance by reversing PD-L1 upregulation in HCC (260).

Collectively, probiotics’ efficacy in enhancing immunotherapy hinges on three core principles: (1) strain-specific metabolite production (e.g., *L. reuteri*’s Trp derivatives, *A. muciniphila*’s bile acid modulations) that aligns with tumor immune needs; (2) activation of conserved immune pathways (cGAS/STING, Th1 polarization) that bridge gut and tumor immunity; and (3) context dependency (tumor type, baseline microbiota) that necessitates precision. Translational progress requires addressing key barriers: standardized probiotic formulations to ensure consistent metabolite output (262), biomarker-guided patient stratification (e.g., matching *A. muciniphila* to HCC patients with dysregulated bile acid metabolism, 261), and avoiding off-target effects (e.g., preventing T cell suppression from mismatched strains, 260). By grounding probiotic use in microbial-metabolite-immune crosstalk rather than generic “microbiota modulation,” these approaches can transform probiotics from adjuncts to precision tools in cancer immunotherapy.

### 5.2 Prebiotics, synbiotics and postbiotics: metabolite-mediated enhancement of cancer immunotherapy

Prebiotics, synbiotics, and postbiotics represent microbiome-targeted strategies that enhance cancer immunotherapy efficacy by reshaping gut microbial composition and reprogramming microbial metabolite profiles—particularly SCFAs, Trp derivatives, and bile acids—which in turn modulate systemic and intratumoral immunity (263). Their therapeutic potential lies in leveraging diet-microbiota-metabolite crosstalk to overcome ICI resistance, though clinical translation is constrained by interindividual microbial heterogeneity, inconsistent metabolite production, and the need for context-specific targeting (263).

Prebiotics, as fermentable dietary components, drive the expansion of metabolite-producing commensals to amplify immunostimulatory signals. For example, pectin supplementation in CRC patient-derived microbiota-humanized mice enhances anti-PD-1 efficacy by enriching butyrate-producing bacteria (e.g., *Faecalibacterium prausnitzii*), with butyrate directly recruiting CD8^+^ T cells to the TME and suppressing Treg differentiation (264). Similarly, dietary resistant starch (a well-characterized prebiotic) elevates colonic SCFA levels (acetate, propionate, butyrate) and enriches *Bifidobacterium* and *Roseburia* in human cohorts; however, its efficacy in boosting ICI responses varies significantly across individuals due to baseline microbiota composition—highlighting the need for personalized prebiotic regimens (228, 265). Postbiotics, defined as microbial metabolites or cell-free extracts, complement prebiotics by delivering direct immunomodulatory activity: soybean-derived postbiotics (enriched in isoflavones and SCFA precursors) suppress colon and lung tumor growth in xenograft models when combined with PD-1 blockade, likely by restoring gut barrier integrity and reducing pro-inflammatory cytokine (TNF-α, IL-6) production (266).

Synbiotics and engineered microbial consortia address the limitations of single-strain or single-prebiotic approaches by combining rationally selected microbes with metabolite-enhancing substrates. A landmark example is Microbial Ecosystem Therapeutic 4 (MET4), a first-in-class 30-strain formulation enriched in ICI-responsive species (e.g., *A. muciniphila*, *Bacteroides vulgatus*). In the MET4-IO clinical trial, MET4 combined with ICIs demonstrated safety in advanced solid tumors and showed signs of efficacy—including stable disease in 40% of patients—by reprogramming bile acid metabolism (elevating tauroursodeoxycholic acid) and enhancing CD8^+^ T cell infiltration (267). This validates the potential of engineered microbial ecosystems to bypass interindividual variability by standardizing metabolite output.

Natural products further expand this toolkit by interacting with the gut microbiota to modulate metabolite pathways critical for ICI responsiveness. Ginseng polysaccharides, for instance, restore the abundance of *Bacteroides* and *Parabacteroides* in tumor-bearing mice, balancing the Kyn/Trp ratio (reducing immunosuppressive Kyn) and elevating SCFAs to rejuvenate PD-1/PD-L1 blockade efficacy (268). Sea cucumber polysaccharides synergize with anti-PD-1 therapy by shaping the gut microbiota to increase ICA—an AhR ligand that enhances CD8^+^ T cell cytotoxicity while suppressing Tregs—reducing MC-38 tumor burden in mice (269). Similarly, fucoidan (a brown algae-derived polysaccharide) rectifies Trp-glycerophospholipid dysregulation in breast cancer models, enriching *Akkermansia* and *Lactobacillus* to boost IFN-γ production by CD8^+^ T cells and sensitize tumors to ICIs (270). Complementary natural product-derived strategies include barley leaf supplementation (enriching *Bifidobacterium* to produce inosine, which activates PPARγ signaling and attenuates colitis-associated tumorigenesis) (271) and icariin (a flavonoid from *Epimedium*), which not only induces tumor ferroptosis via mitochondrial dysfunction but also synergizes with PD-1 inhibitors by reshaping the gut microbiota to reduce pro-inflammatory *Escherichia coli* and increase SCFA-producing *Ruminococcus* (272). Even engineered natural product formulations, such as ginger-derived nanoparticles, reprogram gut bacterial phospholipase C activity to accumulate DHA—a lipid metabolite that inhibits PD-L1 expression on tumor cells—enhancing anti-PD-L1 efficacy in murine melanoma models (142). These examples underscore the untapped potential of traditional medicine derivatives to modulate the gut microbiota-metabolite-immune axis (273).

Collectively, prebiotics, synbiotics, postbiotics, and natural products enhance immunotherapy through a shared mechanism: they alter gut microbiota composition to tune metabolite profiles (SCFAs, Trp derivatives, bile acids, lipids) that directly regulate immune cell function—from CD8^+^ T cell activation to Treg suppression. However, three critical barriers hinder clinical translation: (1) metabolic heterogeneity, where efficacy depends on baseline microbiota (e.g., prebiotics fail in individuals lacking SCFA-producing bacteria); (2) formulation reproducibility, particularly for multi-strain consortia (e.g., batch-to-batch variability in MET4’s metabolite output); and (3) lack of predictive biomarkers to stratify patients likely to benefit (e.g., identifying individuals with dysregulated Kyn/Trp ratios who would respond to ginseng polysaccharides). Addressing these barriers will require integrating microbiota sequencing, metabolite profiling, and immune monitoring into clinical trials—ensuring that these strategies move beyond preclinical promise to deliver consistent, personalized benefits in combination with ICIs.

### 5.3 ATBs as double-edged microbiome modulators: metabolite-mediated impacts on cancer immunotherapy efficacy

The role of ATBs in cancer immunotherapy embodies a context-dependent paradox, where their ability to alter gut and tumor-associated microbiota directly reshapes microbial metabolite profiles—ultimately dictating immunostimulatory or immunosuppressive outcomes. Mechanistically, this duality is rooted in ATB-driven shifts in metabolite production that tune immune cell function across tissue-specific TMEs. In pancreatic cancer, for example, gut bacterial translocation fosters an immunosuppressive TME characterized by reduced CD8^+^ T cell infiltration, expanded MDSCs, and impaired macrophage antigen presentation—changes linked to depleted microbial metabolites like IPA and butyrate (274). Paradoxically, targeted ATB treatment in pancreatic cancer models reverses this by restoring immunogenic metabolite balance: it enhances intratumoral T cell activation, upregulates PD-1 expression on effector T cells, and synergizes with ICIs—suggesting ATBs can uncouple microbiota-driven immunosuppression from metabolite-mediated T cell priming (274). This tissue-specific duality extends to cutaneous squamous cell carcinoma, where prolonged broad-spectrum ATB administration depletes pro-inflammatory microbiota (e.g., *Staphylococcus aureus*) and their toxic metabolites, thereby reducing Treg recruitment and enhancing ICI efficacy (275). Beyond ICIs, ATBs can potentiate adoptive cell therapy: vancomycin, by selectively eliminating Gram-positive commensals, expands systemic CD8α^+^ DCs and boosts IL-12p70 production—changes tied to increased microbial Trp metabolism and IPA accumulation—which enhances the antitumor activity of CAR-T cells and γδ T cells in lymphoma and melanoma models (111, 276, 277).

Conversely, untargeted broad-spectrum ATBs compromise immunotherapy efficacy by disrupting microbial metabolite networks critical for immune homeostasis. Gut microbiota depletion following ATB use reduces α-diversity and suppresses production of SCFAs (e.g., butyrate), leading to expanded Treg populations and impaired CD8^+^ T cell cytotoxicity in melanoma patients (278). This metabolite disruption extends to CAR-T therapy: ATB-induced loss of gut microbiome metabolic output (e.g., reduced inosine and IPA) correlates with diminished CD19-CAR-T efficacy in B cell malignancies, as metabolites like inosine are required to sustain CAR-T cell proliferation and IFN-γ production (279, 280). Clinically, these effects translate to worse outcomes: ATB administration within 60 days before or after ICI initiation correlates with reduced OS in NSCLC, urothelial carcinoma, and melanoma, with elderly patients—who often have more vulnerable microbiomes—experiencing particularly poor OS (281–285). ATBs also increase irAEs by disrupting metabolite-mediated gut barrier integrity, allowing pro-inflammatory bacterial translocation (281, 286). Importantly, temporal context mitigates these risks: ATB use preceding chemoimmunotherapy by extended intervals (e.g., >60 days) shows no impact on efficacy, and some earlier negative associations may reflect indication bias (e.g., ATB use as a marker of advanced disease) rather than direct biological causality (287, 288).

Innovative engineering strategies are now addressing ATBs’ off-target effects by enabling selective microbial targeting. For instance, liposomal formulations like liposome-encapsulated silver-tinidazole complex (LipoAgTNZ) specifically eliminate tumor-associated bacteria (e.g., *Fusobacterium nucleatum* in CRC) while preserving gut commensal ecology. This targeted approach releases microbial neoantigens from killed bacteria and restores tumor metabolite balance (e.g., reducing immunosuppressive Kyn), activating CD8^+^ T cell responses that synergize with ICIs (289).

Collectively, ATBs modulate immunotherapy efficacy by reshaping microbial metabolite landscapes: their beneficial effects (e.g., enhancing ICI/ACT in pancreatic cancer and melanoma) arise from selective depletion of pro-tumor microbiota and restoration of immunostimulatory metabolites (IPA, butyrate, IL-12-inducing metabolites), while their detrimental effects stem from broad disruption of metabolite networks that sustain anti-tumor immunity. Translational success requires precision: strategies like targeted ATB formulations (289) or timing adjustments (avoiding ATB use near ICI initiation) balance antimicrobial activity with microbiome preservation, ensuring metabolite-mediated immune homeostasis is maintained. This framework resolves ATBs’ paradoxical role by grounding their effects in metabolite-dependent immune regulation, highlighting the need to integrate microbial metabolite profiling into ATB and immunotherapy regimens.

### 5.4 FMT: restoring microbiome-metabolite balance to overcome immunotherapy resistance

FMT emerges as a powerful strategy to reverse ICI resistance and mitigate treatment-related toxicities by restoring the gut microbiome’s ability to produce immunomodulatory metabolites—addressing the core microbial-metabolite-immune axis dysregulated in refractory cancers. Mechanistically, FMT’s efficacy hinges on transferring metabolite-producing commensal communities from ICI-responsive donors to recipients, thereby reestablishing key metabolic signals that prime anti-tumor immunity. Preclinically, FMT from ICI-responding patients reprograms the gut microbiota of germ-free or ATB-treated mice not only by expanding beneficial taxa (e.g., *Bacteroides thetaiotaomicron*, *Faecalibacterium prausnitzii*) but also by restoring critical metabolites: SCFAs (butyrate, acetate) that enhance CD8^+^ T cell infiltration into the TME and suppress Treg differentiation, and Trp derivatives (IPA) that activate the AhR to boost DC maturation (261, 290, 291). This metabolite-mediated reprogramming translates to enhanced tumor control, with FMT-treated mice exhibiting reduced MDSC accumulation and increased IFN-γ production by intratumoral T cells, synergizing with anti-PD-L1 therapy (290).

Clinically, FMT has demonstrated translational value across malignancies, with efficacy tightly linked to metabolite restoration. In metastatic melanoma, phase I trials (NCT03341143, NCT03353402) show that responder-derived FMT reverses PD-1 resistance in ~20% of refractory patients by reshaping the gut microbiota to produce SCFAs and bile acid derivatives (e.g., tauroursodeoxycholic acid) that reactivate exhausted CD8^+^ T cells and reduce intratumoral PD-L1 expression (126, 292). A multicenter phase I trial (NCT03772899) further confirms that FMT from healthy donors, combined with ICIs as first-line therapy for melanoma, is safe and associated with increased serum butyrate levels, which correlate with longer PFS (293). Beyond melanoma, FMT restores ICI sensitivity in treatment-refractory esophageal cancer and HCC (NCT04264975) by normalizing the Kyn/Trp ratio—reducing immunosuppressive Kyn while increasing immunostimulatory IPA—and balancing bile acid metabolism (294). In NSCLC, FMT delays tumor progression by enriching *Enterococcus faecalis* and elevating SCFAs (butyrate, acetate, caproate), which enhance DC-mediated antigen presentation and suppress MDSC function (295).

Notably, FMT also mitigates ICI-related toxicities (e.g., colitis) by restoring microbiota-mediated metabolite homeostasis: it rebalances anti-inflammatory SCFAs and reduces pro-inflammatory bacterial metabolites (e.g., lipopolysaccharide), thereby calming excessive mucosal immune activation (296, 297). This dual ability to resensitize tumors to ICIs and alleviate toxicities positions FMT as a multimodal tool in precision oncology (298).

Critical barriers to widespread clinical adoption remain, however, and are inherently tied to metabolite consistency: donor-recipient compatibility (e.g., matching donors with high SCFA-producing microbiota to recipients with depleted SCFA levels), variable microbial engraftment (which impacts metabolite output), lack of standardized FMT processing protocols to preserve metabolite-producing taxa, and long-term monitoring of metabolite-driven immune effects. Resolving these challenges—through biomarker-guided donor selection (e.g., metabolite profiling) and standardized manufacturing—will be essential to realizing FMT’s potential as a metabolite-centric strategy to personalize immunotherapy.

### 5.5 Engineered bacteria: metabolite-directed reprogramming of the tumor immune microenvironment

Genetically engineered microorganisms represent a precision strategy to enhance cancer immunotherapy by delivering or modulating key microbial metabolites directly within the TME, thereby overcoming the limitations of systemic metabolite administration and inconsistent microbiome-driven metabolite production. These engineered strains are rationally designed to produce immunostimulatory metabolites, deplete immunosuppressive TME metabolites, or remodel microbial metabolism to restore anti-tumor immunity—with efficacy tightly linked to their ability to tailor metabolite profiles *in situ*.

A paradigmatic example is *E. coli* Nissle 1917 (EcN), a probiotic strain engineered for targeted metabolite production. SYNB1891, an EcN derivative, is programmed to synthesize c-di-AMP—a bacterial nucleotide metabolite that activates the STING pathway in DCs. This metabolite-driven STING activation primes DC maturation and enhances cross-presentation of tumor antigens, leading to robust CD8^+^ T cell infiltration and antitumor immunity in preclinical models (225). Similarly, EcN strains engineered by Canale et al. metabolize intratumoral ammonia (an immunosuppressive TME byproduct) into L-Arg, a critical metabolite for T cell proliferation and cytotoxic function. By restoring L-Arg levels, these microorganisms synergize with PD-L1 blockade, as L-Arg repletion overcomes T cell exhaustion and enhances the efficacy of checkpoint inhibition (299). Tumas et al. further modified EcN to co-express IL-2 and leverage its inherent LPS production, which enhances IFN-γ secretion by cytotoxic T cells, while engineered IL-2 amplifies this effect by expanding effector T cell populations—creating a dual metabolite-cytokine synergy (300). EcN’s versatility is further demonstrated by strains reprogrammed to overproduce butyrate (301–303), thereby reinforcing effector T cell function in the TME.

Beyond *E. coli*, engineered *Clostridium butyricum* (L-Trp CB) targets the TME by colonizing hypoxic tumor regions to deliver two complementary metabolite-mediated effects: it produces butyrate to inhibit IDO—an enzyme that depletes Trp to generate immunosuppressive Kyn—and releases Trp to fuel CD8^+^ T cell metabolic demands. This dual action restores the Trp/Kyn balance, reversing T cell exhaustion and sensitizing tumors to immunotherapy (304). Other TME-reprogramming engineered microorganisms address metabolite-driven immunosuppression: photosynthetic bacteria (LAB-1) metabolize excess lactate (a TME metabolite that inhibits T cell function) into pyruvate, reducing lactate-mediated immune suppression and restoring CD8^+^ T cell cytotoxicity (305). Similarly, *Enterococcus* strains engineered to express SagA (a peptidoglycan-modifying enzyme) produce modified peptidoglycan metabolites that activate NOD2 signaling in DCs, enhancing their ability to present antigens and prime Th1 responses—strengthening ICI efficacy (135).

*Salmonella*-based engineered microorganisms exemplify precision metabolite control to balance efficacy and safety. Attenuated *Salmonella typhimurium* strains with controlled LPS production (306) limit systemic toxicity while retaining LPS’s ability to activate TLR4 in the TME—LPS triggers DC maturation and IL-12 production, boosting CD8^+^ T cell recruitment. A further advancement is the SAM-FC strain, an attenuated *S. typhimurium* engineered to co-express cytolysin A (ClyA) and *Vibrio vulnificus* flagellin B (FlaB); while ClyA and FlaB enhance immune infiltration, the strain’s modified metabolic profile (e.g., reduced pro-inflammatory metabolite production) prevents systemic toxicity, ensuring TME-restricted metabolite-mediated immunity (307).

Collectively, engineered bacteria enhance immunotherapy by targeting three core metabolite-mediated processes in the TME: (1) producing immunostimulatory metabolites (c-di-AMP, L-Arg, butyrate) to activate effector immune cells; (2) depleting immunosuppressive metabolites (ammonia, lactate, Kyn) to reverse T cell exhaustion; and (3) modifying bacterial metabolites (peptidoglycan, LPS) to balance activation and safety. Critical translational barriers persist, however, and are rooted in metabolite delivery precision: ensuring engineered bacteria colonize only the TME to avoid off-target metabolite production, optimizing attenuation to prevent immunogenicity while preserving metabolite synthesis, and standardizing manufacturing to maintain consistent metabolite output. Resolving these challenges will enable engineered bacteria to fulfill their potential as metabolite-directed tools for personalized cancer immunotherapy, unifying microbial engineering with immune metabolism.

### 5.6 Dietary regulation: tuning the gut microbiota-metabolite axis to enhance immunotherapy efficacy

Dietary regulation shapes cancer immunotherapy outcomes by reprogramming the gut microbiota’s metabolic output—specifically, by enhancing production of immunostimulatory metabolites or reducing immunosuppressive ones—with efficacy rooted in the causal link between dietary components, microbial metabolism, and effector immune cell function. This “diet-microbiota-metabolite-immune” axis unifies diverse dietary strategies, as each intervention modulates microbial flux toward metabolites that activate conserved anti-tumor pathways (e.g., HDAC inhibition, AhR signaling, STING activation) while mitigating metabolite-driven immunosuppression.

Fermentable dietary fiber exemplifies this axis: fiber is broken down by commensals like *R. intestinalis* and *F. prausnitzii* to produce SCFAs—butyrate, propionate, and acetate—that directly enhance ICI efficacy. Butyrate, in particular, activates cytotoxic CD8^+^ T cells by inhibiting HDAC, which upregulates IFN-γ and granzyme B production (148); in CRC, *R. intestinalis*-derived butyrate correlates with improved anti-PD-1 responses by increasing intratumoral CD8^+^ T cell infiltration (148). Propionate, meanwhile, enriches PD-1^+^CD4^+^ T cells in tumors and upregulates T cell activation markers (CD69, CD25) when combined with PD-1 blockade—effects lost in germ-free mice or those lacking SCFA-producing microbiota (259). Clinically, higher fiber intake (≥20 g/day) correlates with longer PFS in ICI-treated melanoma patients, with fecal propionate levels mediating this association (259), resolving preclinical-clinical alignment. Fiber also modulates Trp metabolism: it suppresses microbial Kyn production while promoting synthesis of ILA and IPA by *Lactobacillus* and *Bifidobacterium* (308), which activate AhR in DCs to enhance antigen cross-presentation (138).

Beyond fiber, other dietary components drive SCFA production through distinct microbial metabolic routes: protein-rich diets and valine supplementation, for instance, promote microbial synthesis of isobutyrate—another immunostimulatory SCFA—that enhances ICI efficacy by boosting CD8^+^ T cell cytotoxicity and reducing Treg accumulation in the TME (140). Specific whole-food interventions further reinforce this axis: spinach consumption drives multi-omics shifts in gut microbiota, enriching taxa that upregulate linoleic acid metabolism and butyrate production while downregulating oncogenic pathways (e.g., Wnt/β-catenin) in CRC models (309)—linking plant-based dietary components to metabolite-mediated tumor suppression.

Dietary Trp further amplifies this effect: Trp-enriched diets provide substrate for intratumoral *L. reuteri* to produce ILA, thereby reversing PD-1 resistance in melanoma models (113). This mechanistic link is reinforced by germ-free studies, where Trp’s benefits vanish without *L. reuteri* colonization (113). Conversely, high-fat or high-cholesterol diets disrupt the axis by altering gut microbiota composition—reducing SCFA-producing taxa and increasing bile acid-metabolizing bacteria (e.g., *Clostridium* spp.). This shifts metabolite profiles toward immunosuppressive bile acids (TCA) and reduced IPA, promoting MDSC accumulation and impairing hepatic immunosurveillance in non-alcoholic fatty liver disease-related hepatocellular carcinoma (NAFLD-HCC) (243, 310). Low-fat diets reverse these effects by restoring *Bifidobacterium* and IPA levels, sensitizing tumors to anti-PD-1 therapy (310).

Sodium chloride (high-salt diet, HSD) exhibits context-dependent modulation of the axis: HSD suppresses MDSC function via p38/MAPK-NFAT5 signaling, expanding cytotoxic NK cells and enhancing CD8^+^ T cell effector function (IFN-γ, granzyme B) to boost anti-PD-1 efficacy in melanoma and CRC models (311, 312). HSD also inhibit Treg suppression while promoting Th1 phenotypes (313) and enhance CD8^+^ T cell effector function (314). However, HSD’s therapeutic application requires careful risk mitigation due to adverse effects including exacerbated irAEs with anti-CTLA-4 therapy (315), reduced *Lactobacillus*/butyrate with disrupted intestinal homeostasis (316), and multi-system toxicity (317–321). This context dependency necessitates personalized dosing—e.g., low-dose HSD (4–6% NaCl) retains immunostimulatory effects without toxicity—or stratification by baseline gut microbiota (e.g., patients with high *Lactobacillus* levels may tolerate HSD better).

Collectively, dietary regulation enhances immunotherapy by targeting two core metabolite-mediated processes: (1) increasing microbial production of immunostimulatory metabolites (SCFAs, ILA/IPA) that activate CD8^+^ T cells, DCs, and NK cells via HDAC inhibition or AhR/STING signaling; (2) reducing immunosuppressive metabolites (TCA, Kyn) by restricting substrates for pro-tumor microbiota. Key translational barriers—interindividual variability in microbiota composition, dietary metabolite thresholds, and toxicity risks—can be addressed via: (1) biomarker-guided stratification (e.g., fecal SCFA/IPA levels to select fiber responders); (b) contextual dosing (e.g., HSD adjusted for gut *Lactobacillus* abundance); and (c) combinatorial diets (e.g., fiber + Trp) to synergize metabolite production. By grounding dietary strategies in microbial metabolite flux rather than isolated food components, this framework resolves fragmentation and provides actionable, mechanism-driven nutritional interventions for precision immunotherapy.

### 5.7 Nanotechnology: precision control of the microbiota-metabolite axis to boost cancer immunotherapy

Nanotechnology transforms cancer immunotherapy by resolving key limitations of microbial metabolite-based interventions—poor bioavailability, off-target toxicity, inconsistent microbiota crosstalk—via engineered carriers that enable targeted metabolite delivery, controlled release, and selective gut-tumor-microbiome modulation. Its core value lies in unifying nanomaterial design with microbial metabolite biology: nanosystems either deliver immunostimulatory metabolites, trigger *in situ* synthesis, or reshape microbiota to restore metabolite balance, while minimizing systemic side effects.

Arginine and NO—critical for T cell activation—are prime targets. Aromatic aldehyde-modified L-Arg nanoassemblies (ArgNP) synergize with anti-PD-L1 by sustaining L-Arg release, enhancing CD8^+^ T cell function and reducing MDSCs (322). Multifunctional platforms like HN-HFPA generate ROS via photodynamic therapy to disrupt the TME, while co-releasing L-Arg and NO to reverse T cell exhaustion (323). Antigen-capturing stapled liposomes (ACSL) use irradiation to trigger L-Arg-mediated STING activation and systemic abscopal effects (324), and ultrasound-responsive L-Arg@PTX nanodroplets alleviate hypoxia to potentiate chemoimmunotherapy (325). Sustained L-Arg delivery via liposomes further remodels the immunosuppressive TME and reverses ICI resistance (326). For bile acid modulation, polyoxazolines-based nanocarriers deliver obeticholic acid to regulate hepatic bile acid balance, reducing immunosuppressive TCA and enhancing intrahepatic CD8^+^ T cell infiltration in HCC (327).

Bacterial outer membrane vesicles (OMVs)—natural tumor-targeting nanocarriers—are engineered for safer immunostimulation (328). ΔpalΔlpxM *E. coli* OMVs activate γδ T cells via phosphoantigen presentation (329), while pyroptosis-inducing OMVs reduce Tregs (330). OMV-nanoparticle hybrids (MV-NPs) improve stability for combination therapies (331), and LPS-modified nanoparticles (LPS-NP) boost ICI efficacy with reduced toxicity (227).

Microbiota-targeted nanosystems include near-infrared-activated nano-engineered *Limosilactobacillus reuteri* (LR-S-CD/CpG@LNP) that modulates Trp metabolism to elevate immunostimulatory I3A (332), prebiotic-encapsulated probiotic spores that enhance SCFA production (333), and plant-derived exosome-like nanoparticles (ELNs) that activate AhR via indoles to reinforce gut barriers (334). For pro-tumor bacteria (e.g., *F. nucleatum*), mimetic nanocarriers selectively eliminate intratumoral bacteria via membrane-fused liposomes (335), while biomimetic systems deliver antibiotics locally without disrupting commensals (336).

Nanotechnology addresses three core challenges of metabolite-based immunotherapy: (1) targeted delivery to the TME/gut; (2) controlled release to maintain therapeutic metabolite levels; (3) selective microbiota modulation. Key barriers—non-target accumulation, batch variability, nanomaterial immunogenicity—are mitigated by biomimetic design (e.g., OMVs, bacterial-mimicking carriers). By grounding nanosystem function in microbial metabolite biology, this framework resolves fragmentation and positions nanotechnology as a precision tool for clinical microbiota-metabolite-immune axis targeting.

## 6 Future research directions and challenges

### 6.1 Mechanistic exploration: deciphering dual roles and context-specific signaling

The dual immunomodulatory effects of microbial metabolites—such as butyrate enhancing CD8^+^ T cell cytotoxicity while suppressing DC function—and their receptor-specific mechanisms (e.g., SCFAs via GPR41/43, Trp derivatives via AhR/IDO1) demand systematic resolution. Emerging tools like single-cell multi-omics and spatial metabolomics, including desorption electrospray ionization mass spectrometry imaging, can map spatiotemporal metabolite dynamics within tumor-immune niches. Conditional knockout models, such as myeloid-specific *Gpr43* deletion, will clarify tissue-specific effects like MDSC-mediated immunosuppression. Additionally, biphasic dose responses necessitate pharmacokinetic-pharmacodynamic modeling to define therapeutic windows. A systems immunology approach integrating metabolomics, immune cell atlasing, and computational modeling is critical to reconcile conflicting evidence and establish predictive frameworks.

### 6.2 Clinical translation: overcoming personalized and technological hurdles

Translating microbiome-metabolite insights requires breakthroughs in precision intervention and technological innovation. Artificial intelligence-driven platforms merging metagenomic, metabolomic, and clinical data may predict patient-specific ICI responses. Engineered probiotics and tumor-targeted nanoparticles offer localized efficacy with minimized off-target effects. However, challenges like post-FMT colonization resistance and inconsistent biomarker performance persist. Multicenter trials are needed to validate surrogate endpoints such as serum IPA/Trp ratios and standardize microbiome modulation protocols.

### 6.3 Ethical, regulatory, and technological synergy

Ethical dilemmas, such as patent disputes over synthetic microbial consortia, highlight the need for open-source strain repositories to democratize access to microbiome therapies. Regulatory harmonization is equally urgent, given divergent Food and Drug Administration (FDA)/European Medicines Agency (EMA) classifications of live biotherapeutics, which impede global trial design. International alliances consortium could unify safety and efficacy standards. Future progress hinges on integrating spatial metabolic imaging with synergistic therapies (e.g., HDAC inhibitors combined with pentanoate) and advancing clustered regularly interspaced short palindromic repeats (CRISPR)-edited probiotics to transform microbiome modulation from serendipitous discovery to precision design.

### 6.4 Toward precision microbiome immunology

Future priorities must focus on rigorous clinical validation of microbiota-targeted interventions, including standardized trials to assess the safety and feasibility of FMT and engineered strains. Addressing interindividual variability through pharmacomicrobiomics will optimize personalized dosing. Deep phenotyping via longitudinal multi-omics profiling, coupled with machine learning, is essential to decode host-microbe-metabolite crosstalk. Strategies to enhance ecological resilience, such as prebiotic scaffolds (e.g., resistant starch), are critical to stabilize therapeutic microbiota engraftment in dysbiotic environments. By bridging mechanistic ambiguity, clinical variability, and ethical-regulatory gaps, microbial metabolites may evolve from adjunctive modifiers to cornerstone therapeutics, ultimately reshaping oncology through precision microbiome engineering.

## Glossary

A2aR: Adenosine A2a receptor
ACSS: Acetyl-CoA synthetase
ACT: Adoptive cell transfer
ADP: Adenosine diphosphate
AhR: Aryl hydrocarbon receptor
AKT: Protein kinase B
ALDH1A3: Aldehyde dehydrogenase 1 family member A3
ALPK1: ADP-heptose activates alpha kinase 1
AMP: Adenosine monophosphate
APC: Antigen-presenting cell
Arg1: Arginase 1
ATB: Antibiotic
ATP: Adenosine triphosphate
CA: Cholic acid
CARs: Synthetic receptors
CAR-T: Chimeric antigen receptor T cell
CCL: C-C motif chemokine ligand
CCR: C-C chemokine receptor
CD: Cluster of differentiation
CDCA: Chenodeoxycholic acid
c-di-AMP: Cyclic di-adenosine monophosphate
cGAS: Cyclic guanosine monophosphate AMP synthase
CHIP: Hsc70-interacting protein
CHPS: Capsular heteropolysaccharide
COX-2: Cyclooxygenase-2
CPS: Capsular polysaccharides
CRC: Colorectal cancer
CREB: cAMP response element-binding protein
CRISPR: Clustered regularly interspaced short palindromic repeats
CTL: Cytotoxic T lymphocyte
CTLA-4: Cytotoxic T lymphocyte antigen-4
CXCL: C-X-C motif chemokine ligand
CXCR: C-X-C motif chemokine receptor
CYP2E1: Cytochrome P450 2E1
DAT: Desaminotyrosine
DC: Dendritic cell
DCA: Deoxycholic acid
DHA: Docosahexaenoic acid
DNA: Deoxyribonucleic acid
EcN: *E. coli* Nissle 1917
EMA: European Medicines Agency
EMT: Epithelial-mesenchymal transition
EPS: Extracellular polysaccharides
ERK: Extracellular signal-regulated kinase
ESCC: Esophageal squamous cell carcinoma
FDA: Food and Drug Administration
FFAR2: Free fatty acid receptor 2
FGF15: Fibroblast growth factor 15
FMT: Fecal microbiota transplantation
FOXM1: Forkhead box M1
FOXO1: Forkhead box O1
FOXO3: Forkhead box O3
FXR: Farnesoid X receptor
GA: Gallic acid
GAPDH: Glyceraldehyde-3-phosphate dehydrogenase
GCDCA: Glycochenodeoxycholic acid
GEMM: Genetically engineered mouse model
GLCA: Glycolithocholate
GPCR/GPR: G protein-coupled receptor
GPX4: Glutathione peroxidase 4
H3K27: Histone H3 lysine 27
HCC: Hepatocellular carcinoma
HDAC: Histone deacetylase
HIF-1: Hypoxia-inducible factor 1
HLA: Human leukocyte antigen
HSC: Hepatic stellate cell
HSD: High-salt diet
I3A: Indole-3-aldehyde
I3C: Indole-3-carbinol
IAA: Indole-3-acetic acid
ICA: Indole-3-carboxylic acid
ICD: Immunogenic cell death
ICI: Immune checkpoint inhibitor
ICOS: Inducible T-cell co-stimulator
ID2: Inhibitor of DNA binding 2
IDA: trans-3-Indoleacrylic acid
IDO: Indoleamine 2,3-dioxygenase
IFN: Interferon
IFNAR1: Interferon alpha/beta receptor 1
IL: Interleukin
ILA: Indole-3-lactic acid
ILK: Integrin-linked protein kinase
IPA: Indole-3-propionic acid
irAEs: Immune-related adverse events
JAK3: Janus kinase 3
Kyn: Kynurenine
LAG3: Lymphocyte-activation gene 3
L-Arg: L-arginine
LCA: Lithocholic acid
LPS: Lipopolysaccharides
LTA: Lipoteichoic acid
mAbs: Monoclonal antibodies
MAdCAM-1: Mucosal addressin cell adhesion molecule-1
MAPK: Mitogen-activated protein kinase
MD2: Myeloid differentiation protein 2
MDSC: Myeloid-derived suppressor cell
MET4: Microbial Ecosystem Therapeutics 4
mitoROS: Mitochondrial reactive oxygen species
MMP: Matrix metalloproteinase
MSI-H: Microsatellite instability-high
MSI-L: Microsatellite instability-low
MSS: Microsatellite-stable
mTOR: Mechanistic target of rapamycin
MyD88: Myeloid differentiation primary response 88
NAFLD-HCC: Non-alcoholic fatty liver disease-related hepatocellular carcinoma
NFAT: Nuclear factor of activated T cells
NF-κB: Nuclear factor κB
NK: Natural killer
NKT: Natural killer T
NLRC5: NLR family CARD domain containing 5
NO: Nitric oxide
NOD2: Nucleotide-binding oligomerization domain-containing protein 2
NSCLC: Non-small cell lung cancer
OMV: Outer membrane vesicle
OS: Overall survival
PBMC: Peripheral blood mononuclear cell
PD-1: Programmed death-1
PDAC: Pancreatic ductal adenocarcinoma
PD-L1: Programmed death-ligand 1
PERK: Protein kinase R-like endoplasmic reticulum kinase
PFS: Progression-free survival
PGA: Polygalacturonic acid
PGE2: Prostaglandin E2
PI3K: Phosphatidylinositol-3-kinase
Pink1: PTEN-induced kinase 1
PMCA: Plasma membrane Ca²⁺
POSTN: Periostin
PPAR-γ: Peroxisome proliferator-activated receptor gamma
PRR: Pattern recognition receptor
QDPR: Quinoid dihydropteridine reductase
RA: Retinoic acid
RAGE: Receptor for advanced glycation end-products
RCC: Renal cell carcinoma
RORγt: Retinoic acid-related orphan receptor gamma t
ROS: Reactive oxygen species
SASP: Senescence-associated secretory phenotype
SCC: Squamous cell carcinoma
SCFA: Short-chain fatty acid
sIgA: Secretory IgA
SLC7A11: Solute carrier family 7 member 11
STAT: Signal transducer and activator of transcription
STING: Stimulator of interferon genes
TAM: Tumor-associated macrophage
TBX21: T-box21
Tc1: Type 1 cytotoxic T cell
TCA: Taurocholic acid
TCF-1: T-cell factor 1
Tcm: Central memory T cells
TCR: T-cell receptor
TDCA: Taurodeoxycholic acid
TFEB: Transcription factor EB
TGF-β: Transforming growth factor-beta
TGR5: G protein-coupled bile acid receptor 5
TIFA: TRAF-interacting protein with a forkhead-associated domain
TIGAR: TP53-induced glycolysis and apoptosis regulator
TIL: Tumor-infiltrating lymphocyte
TLCA: Taurolithocholic acid
TLR: Toll-like receptor
TMA: Trimethylamine
TMAO: Trimethylamine N-oxide
TME: Tumor microenvironment
TNBC: Triple-negative breast cancer
TNF: Tumor necrosis factor
Tpex: Progenitor-exhausted T cell
TRAF: Tumor necrosis factor receptor - associated factor
Treg: Regulatory T cell
Trp: Tryptophan
T_SCM_: T memory stem cell
UA: Urolithin A
UB: Urolithin B
UBA6: Ubiquitin-like modifier activating enzyme 6
UDCA: Ursodeoxycholic acid
ULK1: Unc-51 like autophagy activating kinase 1
UTMD: Ultrasound targeted microbubble destruction
VEGFA: Vascular endothelial growth factor A
VEGFR2: Vascular endothelial growth factor receptor 2
VM: Vasculogenic mimicry
WNK1: With-no-lysine (K) 1
YBX1: Y-box binding protein 1.
